# A regionally coherent ecological fingerprint of climate change, evidenced from natural history collections

**DOI:** 10.1002/ece3.9471

**Published:** 2022-11-01

**Authors:** James D. M. Speed, Ann M. Evankow, Tanja K. Petersen, Peter S. Ranke, Nellie H. Nilsen, Grace Turner, Kaare Aagaard, Torkild Bakken, Jan G. Davidsen, Glenn Dunshea, Anders G. Finstad, Kristian Hassel, Magne Husby, Karstein Hårsaker, Jan Ivar Koksvik, Tommy Prestø, Vibekke Vange

**Affiliations:** ^1^ Department of Natural History NTNU University Museum, Norwegian University of Science and Technology Trondheim Norway; ^2^ Natural History Museum University of Oslo Oslo Norway; ^3^ Centre for Biodiversity Dynamics, Department of Biology Norwegian University of Science and Technology (NTNU) Trondheim Norway; ^4^ Section of Science Nord University Levanger Norway

**Keywords:** distribution, ecological change, herbarium, long‐term ecology, norway, phenology, warming, zoological collections

## Abstract

Climate change has dramatic impacts on ecological systems, affecting a range of ecological factors including phenology, species abundance, diversity, and distribution. The breadth of climate change impacts on ecological systems leads to the occurrence of fingerprints of climate change. However, climate fingerprints are usually identified across broad geographical scales and are potentially influenced by publication biases. In this study, we used natural history collections spanning over 250 years, to quantify a range of ecological responses to climate change, including phenology, abundance, diversity, and distributions, across a range of taxa, including vertebrates, invertebrates, plants, and fungi, within a single region, Central Norway. We tested the hypotheses that ecological responses to climate change are apparent and coherent at a regional scale, that longer time series show stronger trends over time and in relation to temperature, and that ecological responses change in trajectory at the same time as shifts in temperature. We identified a clear regional coherence in climate signal, with decreasing abundances of limnic zooplankton (on average by 7691 individuals m^−3^ °C^−1^) and boreal forest breeding birds (on average by 1.94 territories km^−2^ °C^−1^), and earlier plant flowering phenology (on average 2 days °C^−1^) for every degree of temperature increase. In contrast, regional‐scale species distributions and species diversity were largely stable. Surprisingly, the effect size of ecological response did not increase with study duration, and shifts in responses did not occur at the same time as shifts in temperature. This may be as the long‐term studies include both periods of warming and temperature stability, and that ecological responses lag behind warming. Our findings demonstrate a regional climate fingerprint across a long timescale. We contend that natural history collections provide a unique window on a broad spectrum of ecological responses at timescales beyond most ecological monitoring programs. Natural history collections are thus an essential source for long‐term ecological research.

## Graphical abstract

In this study we used natural history collections spanning over 250 years, to quantify a range of ecological responses to climate change, including phenology, abundance, diversity, and distributions, across a range of taxa, including vertebrates, invertebrates, plants and fungi, within a single region; Central Norway. Our findings demonstrate a regional climate fingerprint across a long timescale, demonstrating the value of natural history collections to ecological research.

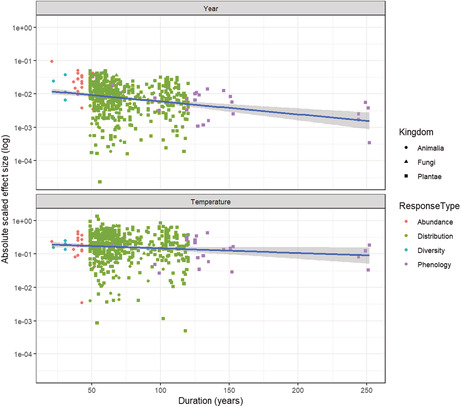

## 1 INTRODUCTION

Climate change has irrefutable, compelling, and wide‐ranging major impacts on ecological systems (IPCC, 2014). Impacts of climate change are apparent across all major habitat types in terrestrial, marine, and freshwater habitats, across taxa from animals, plants, and fungi to microbes (Walther et al., 2002). Climate change alters a range of ecological factors, notably phenology, species abundance, diversity, and distribution. The breadth of ecological responses to climate change leads to ecological fingerprints of climate change across a multitude of taxa within terrestrial (Parmesan & Yohe, 2003; Root et al., 2003), freshwater (Woodward et al., 2010) and marine ecosystems (Poloczanska et al., 2013). Ecological fingerprints of climate change are a suite of responses to a changing climate, apparent across a range of taxa and ecological variables. For example, Parmesan and Yohe (2003) synthesized ecological responses to climate change through global meta‐analyses and found an average shift in distributions poleward by 6 km per decade and phenological advance by 2.3 days per decade. This synthesis was termed a “globally coherent fingerprint of climate change,” since the phenological advance aligned with poleward distribution shifts, and both were consistent with warming temperatures. However, meta‐analyses are known to be susceptible to publication biases, whereby only the studies, which show significant responses to exposure are published, and hence synthesized. Such biases can result from the sampling of climate‐sensitive species or climate‐sensitive locations such as at temperature or moisture extremes (Klesse et al., 2018).

Synthesizing ecological fingerprints of climate change may be challenging. For example, Brown et al. (2016) found that methodological differences accounted for almost three times the variation in species range shifts than the species' ecological shifts and half of the variation in phenological responses. A further complexity is that responses to climate may exhibit threshold effects, rather than simple linear changes (Hillebrand et al., 2020), and these dynamics are rarely accounted for in syntheses. Taken together, sampling biases, methodological differences, and threshold changes imply that identifying ecological fingerprints of climate change may be inaccurate, and this will pose challenges to predict future changes in response to continued climatic change. Furthermore, ecological responses to warming are often lagged behind climate change by decades to centuries (Menéndez et al., 2006), implying that truly long‐term datasets are required to investigate ecological fingerprints of warming.

Natural history collections are an underexploited resource for long‐term ecological research. Natural history collections can be used to quantify a range of ecological responses including distributions, phenology, and species interactions, to multiple drivers of change, including climate change, non‐native species, and pollution, across a large range of taxa and at decadal to centurial scales (Meineke et al., 2019). These temporal scales far outstrip most, if not all, ecological monitoring programs. Since natural history collections are not sampled with an aim of quantifying the impacts of climate change, nor other forms of environmental change, they may be less likely to be susceptible to sampling and publication biases when investigating climate change responses. Most natural history collections are sourced regionally (Bakker et al., 2020), signifying that they have great potential for investigating ecological fingerprints of climate change at a regional scale.

The objective of this study is to identify the level of coherence in ecological change over a centurial timescale across taxa (invertebrates, vertebrates, fungi, and plants), ecosystems (marine, freshwater, and terrestrial), and ecological variables including distributions, diversity, and phenology. We investigate whether ecological responses across a range of taxa, ecosystems, and ecological variables within a single region vary over time and in association with changing temperature. We use natural history collections spanning over 250 years from Central Norway. We test the hypotheses that 1. Ecological variables derived from natural history collections show similar trends over time and with temperature. 2. Longer natural history collection time series show stronger temporal trends and responses to temperature and 3. Breakpoints in the temporal trends in the ecological state (i.e., periods across which the rate of ecological change differs) occur at similar times as breakpoints in the temperature trends.

## 2 METHODS

### 2.1 Study region

**FIGURE 1:**
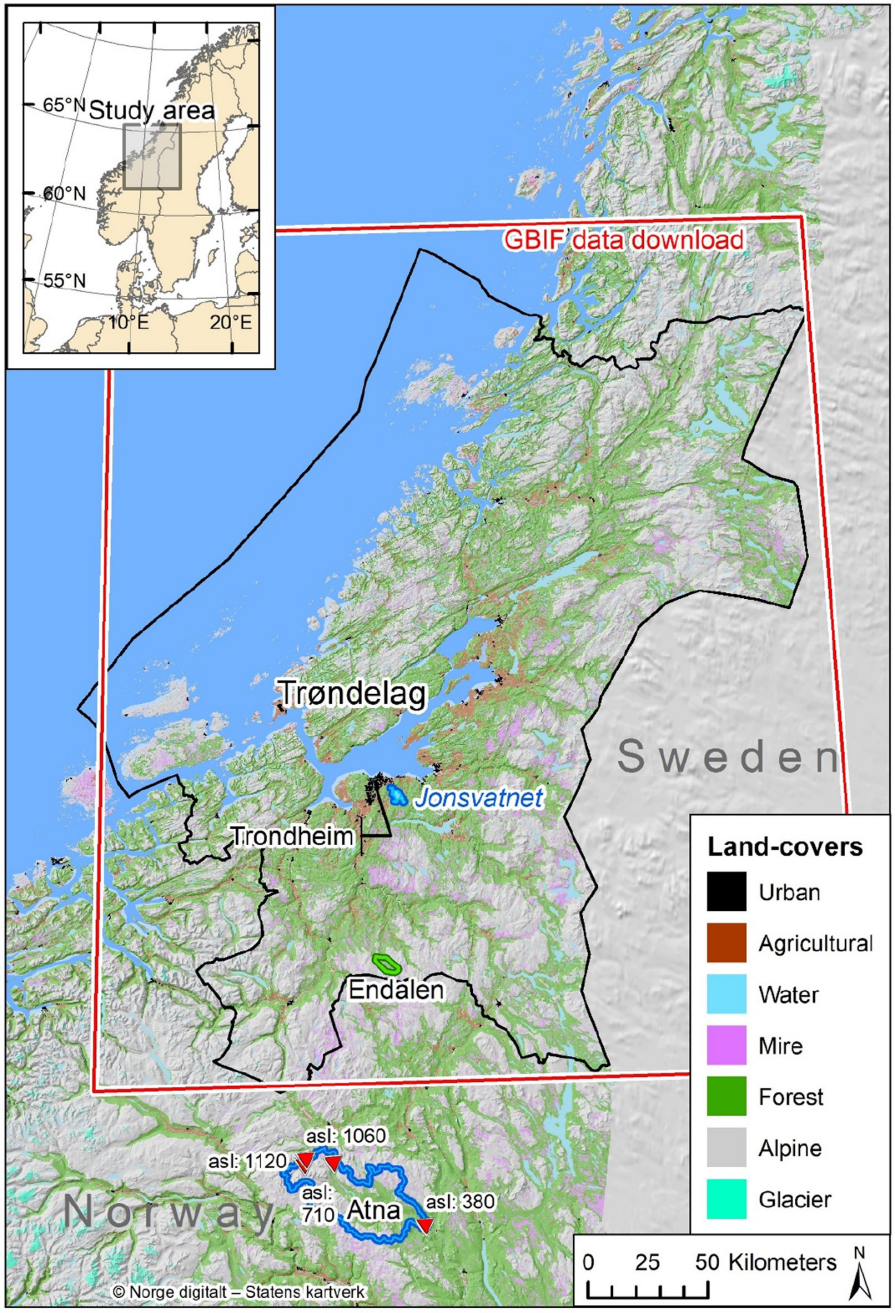
Map of study region, showing major land‐cover types, the county boundary of Trøndelag (black polygon), the geographic limit of the GBIF download (the Norwegian portion of the red bounding box) and the location of specific datasets included in this manuscript (Jonsvatnet, Endalen, and Atna). The study population is the natural history collections of the NTNU University Museum located in Trondheim.

Our study population was the natural history collections belonging to the Department of Natural History at the NTNU University Museum, Norwegian University of Science and Technology, located in the city of Trondheim https://www.ntnu.edu/museum/natural‐history‐collections. These collections contain around 1.4 million specimens from many parts of the world, but the majority (ca. 65%) are from Central Norway. Our study region was defined broadly as Central Norway, of which the county of Trøndelag forms the greater part (Figure 1). The region of Central Norway spans a range of biogeographical gradients, from boreonemoral, through boreal and to high alpine zones, and from highly oceanic to slightly continental sectors (Moen, 1999). Our study region also includes marine ecosystems within the region.

Within our study region, we aimed to quantify a range of ecological responses to climate over time across taxa and natural history collections. A range of ecological parameters potentially responding to climate warming was quantified across our study region, and are termed ecological responses, herein. The ecological responses were selected from the collections of the NTNU University Museum, Department of Natural History, with an aim of covering a broad range of ecological contexts and long time series. The ecological responses included a range of taxa from plants, fungi, and animals, a range of ecosystems within terrestrial, freshwater, and marine habitats, and a range of ecological scales, including phenology, abundance, diversity, and distributions (Table 1).

**TABLE 1:** List over ecological responses and datasets used to characterize these. Responses are ordered by ecological scale, and the same order is used to present the responses across the study.

Dataset	Ecological response	Kingdom	Taxa	Location (Figure 1)	Range of years	Number of years included	Number of species analyzed	Dataset citation/DOI
Herbarium records	Phenology	Plantae	Trachaeophyta (22 species)	Central Norway	1763–2020	153	24	Specimens: https://doi.org/10.15468/zrlqok Flowering data: 10.6084/m9.figshare.17086139
Breeding bird surveys	Abundance	Animalia	Aves, Passeriformes (5 species)	Endalen	1967–2018	50	5	10.6084/m9.figshare.17086217
Limnic zooplankton	Abundance	Animalia	Arthropoda, Rotifera (16 taxa)	Jonsvatnet	1977–2020	40	16	https://doi.org/10.15468/ubpk0i
Benthic mayflies and stoneflies	Diversity	Animalia	Ephemeroptera and Plecoptera	Atna (four sampling localities at different elevations)	1986–2018	32	31	10.6084/m9.figshare.17086277
Marine invertebrates	Distribution	Animalia	Arthropoda, Echinodermata, Mollusca (9 species)	Norwegian waters within bounding box	1906–1981	19	9	https://doi.org/10.15468/ddbs14
Animals	Distributions	Animalia	Annelida, Arthropoda, Mollusca, Chordata (153 species)	Norwegian part of bounding box	1902–2020	114	153	https://doi.org/10.15468/dl.cybszc
Plants	Distributions	Plantae	Bryophyta, Marchantiophyta, Tracheophyta (605 species)	Norwegian part of bounding box	1900–2020	120	605	https://doi.org/10.15468/dl.cybszc
Fungi	Distributions	Fungi	Ascomycota, Basidiomycota (21 species)	Norwegian part of bounding box	1927–2019	58	21	https://doi.org/10.15468/dl.cybszc

### 2.2 Ecological responses

#### 2.2.1 Phenology of plants

To quantify plant phenology, we selected specimens of vascular plants from the Trondheim herbarium (TRH). We downloaded the whole dataset (Norwegian University of Science and Technology, 2021) and subsequently filtered to include only specimens with images from Central Norway (Trøndelag county, the districts Nordmøre and Romsdal of Møre og Romsdal county and district Helgeland of Nordland county). Records without precise dates of collection were excluded. From the resulting dataset, the list of taxa that were prioritized for assessment fulfilled the following criteria: 1. The number of specimens within each species should exceed 150 specimens (specimens inside envelopes were omitted) and cover a period of around 100 years or more. 2. The morphology of the plant should enable easy observation of the phenological parameters. 3. Habitat specialists were prioritized over habitat generalists, as phenotypic plasticity of traits under selection, like phenology, is more restricted in habitat specialists compared with habitat generalists (Van Tienderen, 1997). Among the resulting potential taxa, we then selected species to cover the variation among vascular plants in Central Norway regarding both habitat, growth form, and taxonomic group; thus, the chosen species include representatives from the following groups of nonwoody species: lowland and (sub)alpine species, annuals, perennials, ferns, and grasses and sedges. However, most grasses and sedges were excluded as it was difficult to assess the degree of development of flowers from the photographed specimens. Species known to flower shortly after snow‐melt were also excluded as these could introduce biases in estimates of flowering date due to the limited window for the sampling ages were scored of these.

After these filtering steps, 24 vascular plant species remained for scoring of phenology based on the image files within the GBIF dataset. Phenology of the herbarium specimen images was scored using a modification of the PhenObs protocol (Nordt et al., 2021). For this study, the parameter “flowering intensity” was analyzed, which was scored as the percentage of open flowers in relation to the potential number of flowers, which was the total number of flower buds, flowers, and fruits on the specimen. This was assessed on a single plant or, if the specimen/herbarium sheet contained more than one plant, as a mean. As a measure of flowering timing, we used the earliest date of peak flowering, since this is less susceptible to biases in natural history collections than first flowering (Meineke & Daru, 2021). For each species, peak flowering intensity was assessed as the flowering intensity at or above the 75% quantile of all flowering intensity records for that species. For each year, the earliest date of peak flowering intensity within the dataset was extracted and used as a response variable. For two species, there were fewer than 10 years where peak flowering intensity was recorded; these species were omitted, leaving 22 species.

#### 2.2.2 Abundance of boreal forest breeding birds

Abundance data of boreal forest breeding birds were acquired from surveying a 0.24 km^2^ transect annually from 1967–2018 (except 1998 and 1999). The transect is located in subalpine birch (*Betula pubescens*) woodland at 800–880 m.a.s.l. in Endalen (62°45′ N, 10°30′ E; Figure 1), Central Norway. We include records of 5 songbird species with sufficient abundance (minimum of 6 breeding pairs km^−2^) and suitability for repeated territory counts (i.e., not colonial or nomadic species). The transect was surveyed annually around 10 times per year mainly during morning hours, spread over 3–4 periods when breeding birds in the area are most active (i.e., during June), following a standard procedure described in (Bibby et al., 1992; Thingstad et al., 2015). Within each year, surveys were aggregated based on the clustering of observations from the temporally independent surveys, where territories could be formed around clusters of three or more observations. Clusters of observations on the edge of the transect were regarded as parts of territories (to the nearest quarter of a territory) when included in the density estimate. The density of territories (km^−2^) was used as a response variable.

#### 2.2.3 Abundance of limnic zooplankton

To assess the abundance of limnic zooplankton, we used a dataset (Hårsaker & Daverdin, 2022) sampled within the limnetic zone of the lake Jonsvatnet, Central Norway (63°22′ N, 10°37′ E, Figure 1), 150 m above sea level. Sampling was based on one sample from each of three basins (Store Jonsvatn, Lille Jonsvatn, Kilvatn) within the lake in the years 1977–2020. All samples are taken during the period of May–October. Samples were taken three times in 1977 and 1980, four to six times in 1983–1990, six to nine times in 1991–2016, and seven times in 2017–2020. Zooplankton was sampled with a 1 m long tube sampler. Each sample contained 5 L of water. A vertical column of water extending from 0 to 20 m depth was consistently sampled every 1 m. Samples from 5 m layers were merged. Zooplankton samples were preserved with Lugol's solution in the field. All zooplankton samples were later identified and enumerated in the lab. Counts were carried out on the total sample or on subsamples containing 1/10 of the total sample. Taxa with <100 observations across the three sampling sites were filtered out for further analysis. The average abundance of each species (m^−3^) was used as a response variable.

#### 2.2.4 Diversity of mayflies and stoneflies

The diversity of mayflies and stoneflies was quantified from a long‐term dataset at several elevational levels within the Atna catchment in Rondane (Figure 1). Mayfly and stonefly larvae were collected by Surber sampling or by kick and sweep sampling. The net mesh used in both Surber and kick and sweep sampling was 0.5 mm. During 1987–2002, Surber samples with an area of 0.1 m^2^ were taken 1–4 times a year, each time with 5 replicated samples (Aagaard et al., 2004). Species richness was calculated as the maximum across these replicates. From 2003 on the samples were taken by kick and sweep sampling as a subsample of a single 5‐min period sampling. The number of taxa per sampling station was used as a response variable. To account for the different methods, the method type was fitted as a fixed effect in models of species richness.

#### 2.2.5 Distributions

For the assessment of changes in latitudinal distributions of nonmarine species, we downloaded species occurrence records from the Global Biodiversity Information Facility (GBIF.org, 2021) meeting the following criteria: 1. No geospatial issues; 2. including coordinates; 3. located within a predefined rectangular bounding box encompassing the study area (Figure 1); 4. located in Norway (country = NO; to omit records from Sweden); 5. only records from the NTNU University Museum (institutionCode = ntnu‐vm, NTNU‐VM, trh or TRH). The dataset was further filtered to only include presence records (occurrenceStatus = PRESENT) of species with full binomial species names, collected/observed between the years 1900–2020 to match the temperature data. Only species for which there were at least 50 years between the first and last occurrence record, and species‐year combinations for which at least five records of the species in question had been registered in the specific year to ensure a minimum sampling effort. The R packages used for this were rgbif (Chamberlain & Boettiger, 2017), sf (Pebesma, 2018), and raster (Hijmans, 2016).

For changes in latitudinal distributions of marine invertebrates, records meeting the above 5 criteria were selected from the Marine invertebrate collection (NTNU University Museum; [Bakken et al., 2021]) and filtered as above by removing taxa not identified to species level, with <5 records per year and <50 years of data after 1900. Many taxa were sampled from a single location within and in some cases between years so to ensure a range of locations in Trøndelag were sampled for any given year, records with a range of latitudes of <0.4° (≈44 km N–S) per year were removed, resulting in data from 35 species. It was assumed that cold‐adapted (rather than wide‐ranging) taxa were more likely to show distributional changes in relation to regional warming, so we examined the known distributions of these 35 species on GBIF and retained records from nine species with boreal–arctic distributions, resulting finally between 50 and 76 years of data for each species ranging between 1906–1981.

For each species, the registered latitudes were summarized across all records per year. For every year, the 90th percentile of registered latitudes was calculated and used as the leading edge of the geographic distribution. The 90th percentile was used rather than the northernmost record to avoid undue influence of extreme, single observations. The leading‐edge latitude was modeled for animals, plants, fungi, and marine invertebrates separately.

### 2.3 Temperature data

As a hypothesized driver of terrestrial and freshwater ecological responses, we used air temperature data as an independent variable. Due to the disconnect between air temperature and seawater temperature (Kara et al., 2007), we used a separate temperature data series for marine ecological responses. For terrestrial and freshwater ecological responses we downloaded monthly temperature data for Trøndelag (Norwegian Centre for Climate Services, 2021) covering the period 1900–2020. This consisted of monthly records for all weather stations within Trøndelag county. Annual means were calculated across all stations.

Marine data were extracted from the permanent oceanographic station Bud (Institute of Marine Research, 2021). This is in Møre og Romsdal county (62.9333° N, 6.7833° E) but is the closest, upstream, and most relevant for ocean temperatures in Trøndelag, as well as representing the longest time series. Due to the irregular sampling of water temperatures in time, and across depths, we used the annual maximum temperature recorded at 200 m depth at this station.

### 2.4 Data analysis

Due to nonlinear dynamics, the temperature data were analyzed using two complementary approaches. First, we used segmented regression to identify breakpoints in the slope of temperature against year. Based on visual inspection of the data, we tested for two breakpoints in the relationship, using the p‐score test (segmented package in R; Muggeo, 2008; Muggeo, 2016) with an alternative hypothesis of fewer than two breakpoints. Secondly, we used a general additive model (GAM) to fit a smooth function through the mean annual temperature time series. To investigate the direction of change of the temperature trajectory, we took the first derivative of the GAM fit (i.e., dTemperature/dTime) and plotted this as a time series. GAM models were fit using the *mgcv* package (Breheny & Burchett, 2017), visualized with the *visreg* package (Breheny & Burchett, 2017), and derivative extracted with the *gratia* package (Simpson, 2020).

To test whether each ecological response varied with time (year) and temperature (mean annual temperature) we first estimated linear regression slopes and standard errors for each species (or sampling station) within each ecological response (Table 1). We then used unweighted, fixed‐effects meta‐analytical models within the R package *metafor* (Viechtbauer, 2010) to estimate the overall modeled slope and 95% confidence intervals for each ecological response. We choose to use unweighted models rather than weighting by inverse variance of the slope estimates, since the different estimates within each ecological response were estimates from separate taxa (or sampling stations), rather than independent estimates of the same parameter. For the same reason, we choose to fit each taxon (or station) as a fixed effect rather than a random effect.

To ensure that the effect sizes are interpretable in ecological terms, we used unscaled (raw) response variables to present each ecological effect size in isolation. However, to synthesize the effect sizes across ecological responses, allowing us to address hypothesis 2, we used the absolute effect size estimated using centered and scaled ecological response variables. Centering (on the mean) and scaling (by standard deviation) allow each effect size to be interpreted as standard deviation units from the mean. We used the absolute value to account for the expected differences in direction of ecological responses to climate (e.g., earlier phenology is represented as a negative effect size).

To test whether there were shifts in the slope of the relationship between ecological responses and time, and whether these corresponded with changes in the temporal trends in temperature data, we again used segmented regression. Within each ecological response that spanned 80 years or greater, we tested for one or two significant breakpoints (using the p‐score test; see above). If one or two significant breakpoints existed, the years of these were extracted. We used Kolmogorov–Smirnov test to test whether the distribution significantly differed from a simulated uniform and normal distribution, and a bimodal distribution with peaks at the same years as breakpoints in the temperature data.

## 3 RESULTS

### 3.1 Temperature trends

**FIGURE 2:**
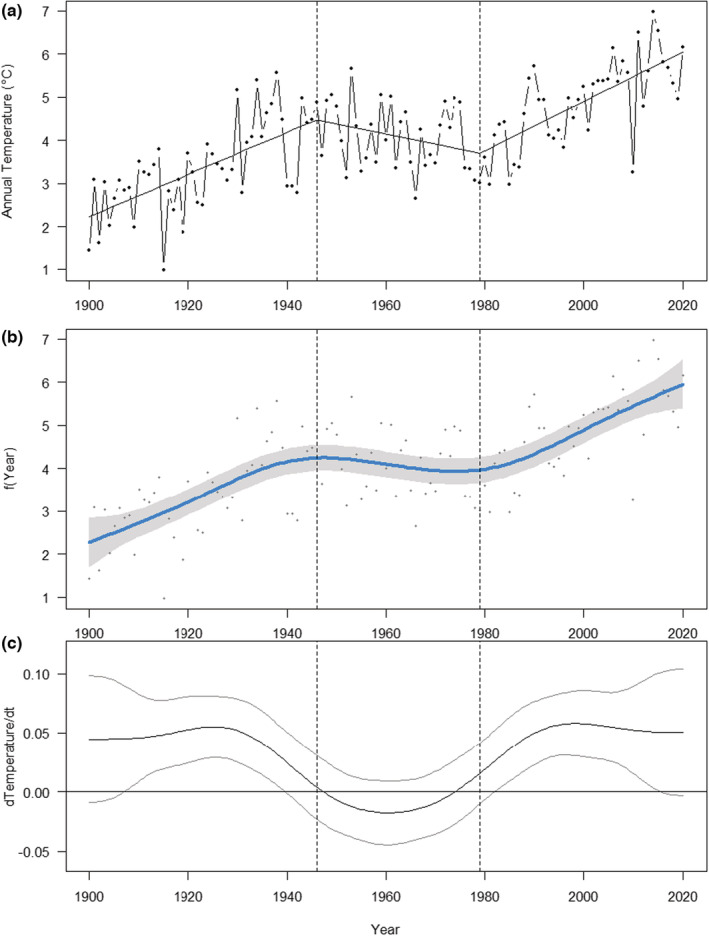
Terrestrial climate data. (a) Annual mean temperature trends from 1900 to 2020 averaged across all climate stations in the county of Trøndelag. Segmented regression fit shown with dotted lines located at years at which significant breakpoints occurred (b) GAM smooth of same data. (c) First derivative (dTemperature/d*t*) of the GAM fit plotted in part. (b) Confidence intervals overlapping with 0 indicate stability.

There were three distinct phases in the trends in mean annual air temperature across our study region (Figure 2). There were two significant breakpoints in the relationship between mean annual temperature and year; in 1946 (standard error 4.8) and 1979 (standard error 4.5; two‐sided p‐score test 18.64, *n* = 100, *p* < .001). During the first phase, there was an increase in mean annual temperature between 1900 and 1946 of an average of 0.05°C (±0.008 standard error) per year. From 1946 to 1979, the temperature showed a slight decrease (−0.02°C year^−1^ ± 0.010 standard error) while between 1980 and 2020, the mean annual temperature again increased by 0.06°C year^−1^ (±0.009; Figure 2a). The three‐period pattern was also supported by the GAM analysis (Figure 2b) and the first derivative of the GAM relationship, which showed a significant positive change at the start and the end of the period, but stability (overlapping zero) during the middle please, rather than cooling (Figure 2b). The marine temperature data were sporadic. There was no trend in maximum marine temperature over time (*F*_1,42_ = 0.61, *p* = .44; Figure S1).

### 3.2 Ecological responses

**FIGURE 3:**
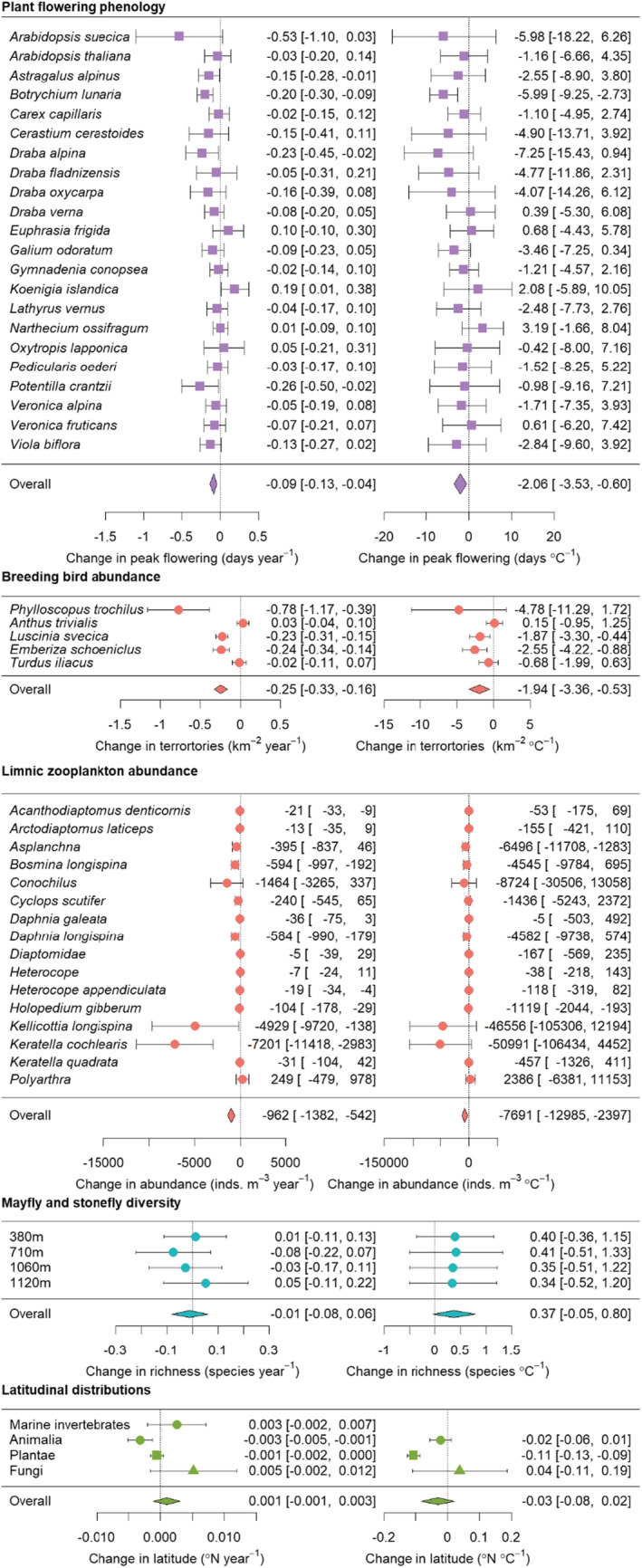
Forest plots showing regression slopes for each dataset and taxon or habitat within each dataset against year (left) and temperature (right). Slope estimates and 95% confidence intervals are shown as points and error bars for individual taxa (or sampling stations). Overall estimates, calculated through unweighted, fixed effects meta‐analytical models are shown by diamond polygons. Point colors and shapes correspond to Figure 4. Due to sporadic marine temperature data, marine invertebrates are only regressed against year. Individual species distribution regression slopes are shown in Table S2.

Across 22 species, plant flowering phenology showed an average advance of 9 days per century (change in earliest date of peak flowering: −0.09 days per year, [95% confidence interval: −0.13, −0.04]; Figure 3). A few species individually showed advances in flowering phenology: namely *Botrychium lunaria, Draba alpina*, and *Potentilla crantzii*. Meanwhile, the earliest date of peak flowering of *Koenigia islandica* became later by 19 days per century [1.9 days year^−1^, CI: 0.01, 0.38]. In terms of temperature, plant flowering phenology advanced by an average of 2.06 days °C^−1^ [CI: −3.53, −0.60] of warming (Figure 3), with the only individual species showing a response deviating from zero being *Botrychium lunaria* (−5.99 days °C^−1^ [CI: −2.73, −9.25]).

The abundance of boreal forest breeding bird territories decreased by 0.25 territories km^−2^ per year [CI: −0.16, −0.33] on average across the five included species. The density of territories of *Phylloscopus trochilus*, *Luscinia svecica*, and *Emberiza schoeniclus* individually decreased, while the territory density of tree pipit and redwing were more stable (Figure 3). There was also an overall decrease in territories when regressed against temperature, with a decrease of 1.94 territories km^−2^ °C^−1^ [CI: −0.53, −3.36].

The abundance of limnic zooplankton decreased with time by 962 individuals m^−3^ year^−1^ [CI: −542, −1382]. Seven individual taxa decreased over time, with particularly high‐magnitude decreases in two rotifer species: *Kellicottia longispina* (−4929 individuals m^−3^ year^−1^ [CI: −138, −9720] and *Keratella quadrata*; −7201 individuals m^−3^ year^−1^ [CI: −2983, −11,418]; Figure 3). The abundance of limnic zooplankton also decreased with mean annual temperature by an average of −7691 individuals m^−3^ °C^−1^ [CI: −59.9, −324.6], but only two taxa; *Asplanchna* and *Holopedium gibberum* individually decreased with temperature.

The species richness of mayflies and stoneflies did not change over time with an average effect of −0.01 species per year [CI: −0.08, 0.06]. With temperature, the change in species richness across the stations was on average 0.37 species °C^−1^, but this did not differ from zero [CI: −0.05, 0.80].

Species distributions on average did not change over time (0.001°latitude year^−1^ [CI: −0.001, 0.003]) or temperature (−0.031°latitude °C^−1^ [CI: −0.083, 0.020]). However, animal distributions decreased in latitude over time (−0.003°latitude year^−1^ [CI: −0.005, −0.001]) while plant distributions decreased in latitude with temperature (−0.108°latitude °C^−1^ [CI: −0.129, −0.087]). Due to the low overlap between marine invertebrate distribution data, and marine temperature data, it was not possible to investigate how marine invertebrate distributions varied with temperature. Effect sizes for individual species are shown in Table S1.

### 3.3 Synthesis of ecological responses

**FIGURE 4:**
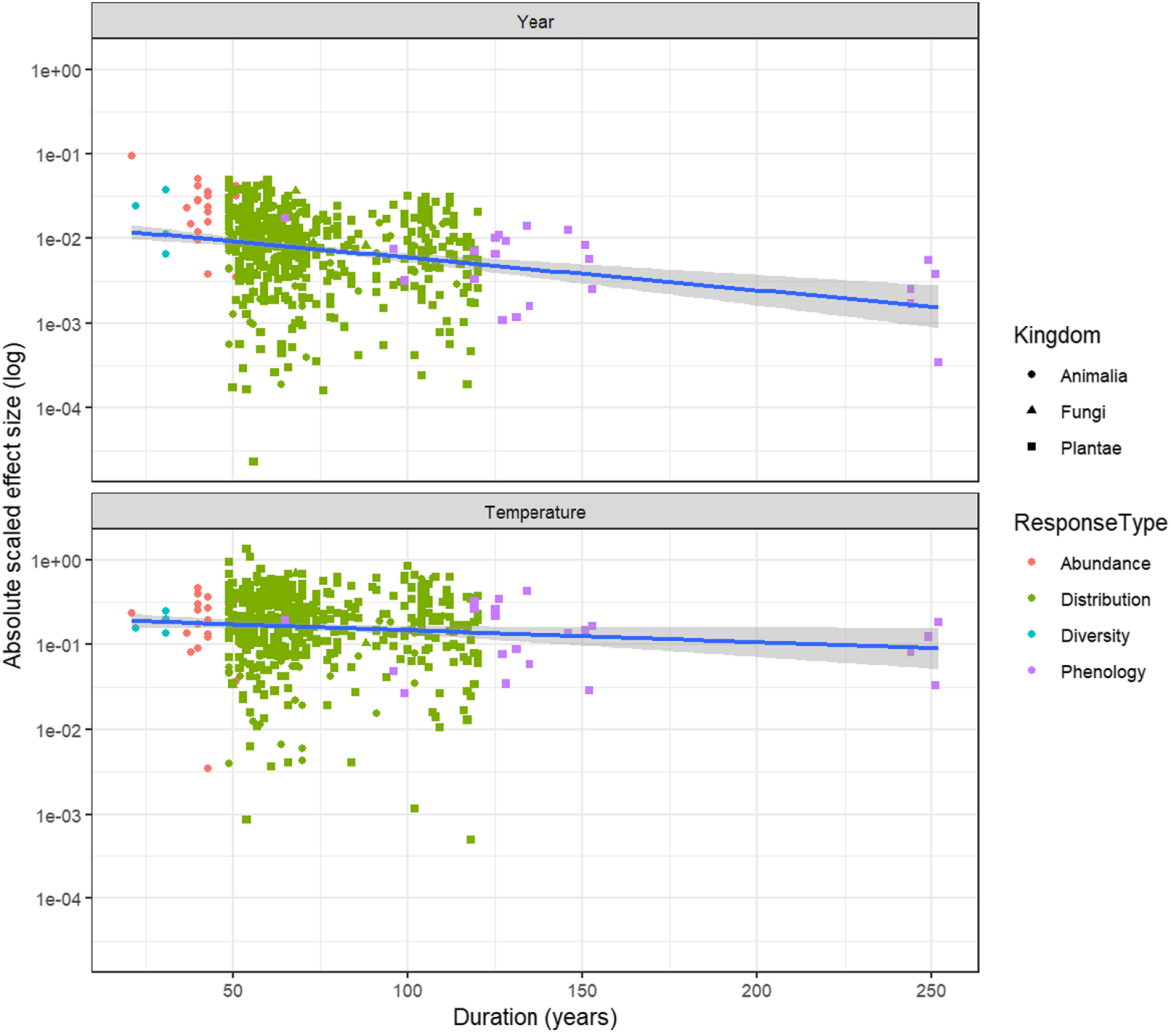
Effect sizes against time (top) and temperature (bottom) (absolute regression slopes, with centered and scaled response variables, log‐transformed response variable), plotted against the duration of the study (x, assessed as the difference between the latest and earliest year with estimate). Point shapes correspond to different taxonomic Kingdoms, and color to the different types of ecological effects quantified.

The log‐transformed absolute scaled effect size of relationships between ecological responses and time, decreased with the duration of the dataset (−0.009 year^−1^ ± 0.002 SE; Figure 4). This relationship was apparent across all ecological responses (*F*_1,546_ = 35.5, *p* < .001) and within the two ecological response types with high variation in response duration (Distributions: slope = −0.007 ± 0.002, *F*_1,499_ = 9.9, *p* = .002 and phenology: slope = −0.008 ± 0.003, *F*_1,20_ = 4.5, *p* = .03). The effect size between ecological response and temperature also decreased with dataset duration (−0.003 ± 0.002 SE; Figure 4), although this relationship was not so strong (*F*_1,537_ = 4.17, *p* = .042), and was not present within either distribution data (*F*_1,490_ = 1.73, *p* = .19) or phenology data (*F*_1,20_ = 0.44, *p* = .52).

**FIGURE 5:**
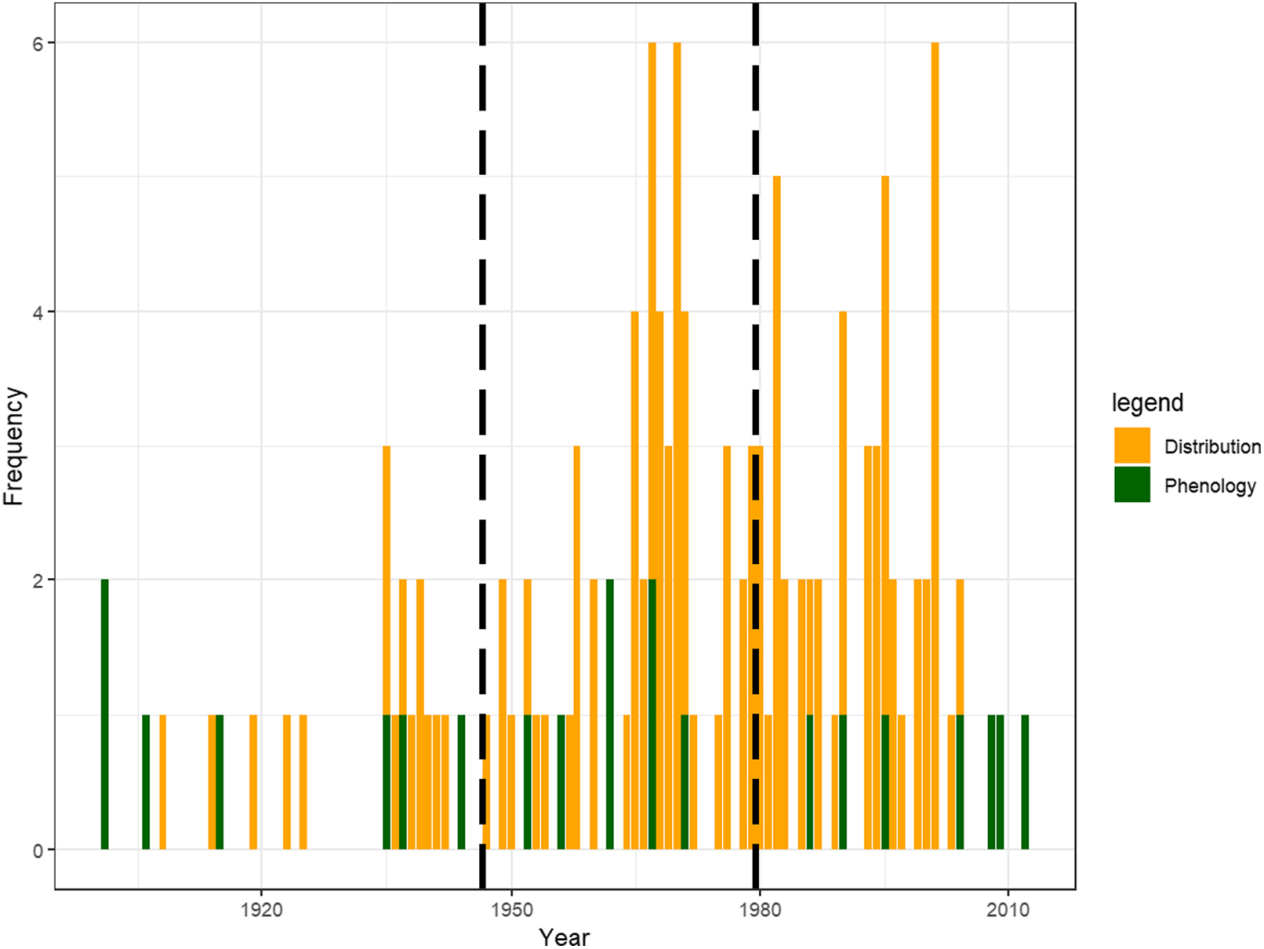
Distribution of breakpoints in linear regressions of ecological responses against time, colored by data type (bars are stacked). Only ecological responses with durations of at least 80 years were included. Vertical dashed lines show the years of breakpoints in the mean annual temperature data (Figure 2).

In total there were 131 breakpoints across the (nonmarine) distribution data and plant phenology data (Figure 5). There were no notable peaks in the distribution of breakpoints around the same periods as the annual temperature temporal trends (1946 and 1979; see Figure 2). Breakpoints were distributed between 1901 and 2012. There were peaks in the late 1960s to 1970s and late 1990s to early 2000s. The distribution of breakpoints deviated from normal (Shapiro test; *W* = 0.949, *p* < .001). It also significantly differed from a uniform distribution (Kolmogorov–Smirnov test two‐sided, *D* = 0.256, *p* < .001) and a simulated bimodal distribution (Kolmogorov–Smirnov two‐sided, *D* = 0.265, *p* < .001). Cumulative distribution functions of these are shown in Figure S2.

## 4 DISCUSSION

By quantifying a range of ecological responses across taxa within a single region, our study documents a regionally coherent fingerprint of climate change on Central Norwegian nature; a fingerprint with higher spatial specificity than at global scales (Parmesan & Yohe, 2003; Root et al., 2003). We found changes have occurred over time, and in relation to temperature. Our results show that the phenology of plants has advanced by 9 days per century, the abundances of boreal forest breeding birds and limnic zooplankton have decreased, but species distributions have been largely stable within the region. Surprisingly, the length of the record had the opposite effect than hypothesized, with longer studies having lower absolute effect sizes for temporal trends. In addition, thresholds in the temporal trends in ecological responses did not correspond to thresholds in temperature trends. Our study demonstrates the importance of natural history collections to synthesize the impacts of environmental change at a regional scale, further extending the known value of natural history collections for ecological science (Meineke et al., 2019).

### 4.1 Phenology

Phenological effects observed in central Norway (average advance of 0.9 days decade^−1^) are similar in direction but generally weaker than observed in studies across Europe (2.5 days decade^−1^; Menzel et al., 2006), southern Germany (1.3–2.1 days decade^−1^; [Renner et al., 2021]) or Britain (4.5 days decade^−1^; Fitter & Fitter, 2002). The advance in flowering per degree warming that we observed in Central Norway of 2 days °C^−1^ is also weaker than in southern Germany of 3.2–4.2 days °C^−1^ (Renner et al., 2021), but comparable with the advance in the flowering of 2.1 days °C^−1^ that Kimball et al. (2014) modeled for three American alpine plant species over a 77‐year period.

The relatively low estimated values from our study are contrary to the expectation of a stronger effect of climate warming at more northern latitudes (Høye et al., 2007). This may be due to our dataset being derived from collected specimens, rather than a designed study. Other studies (e.g., Fitter & Fitter, 2002) also focused on the first flowering dates of early spring‐flowering species, which are likely to be highly plastic, whereas we excluded early spring‐flowering species. First‐flowering date estimates from collected specimens tend to be later than first‐flowering estimates from observations (Davis et al., 2015) due to the opportunistic nature of the herbarium collections relative to observations of flowering that are sought for during a study design. Our study deliberately incorporated data from a range of biogeographical contexts, from boreonemoral, through boreal and to high alpine zones, and from highly oceanic to slightly continental sectors (Moen, 1999), we argue that the result of general flowering advancement over the period, is robust.

Two of the five annuals showed trends contrary to the overall average. The flowering of *Koenigia islandica* became later over time (and in relation to temperature), this species grows in spring‐influenced snow beds along an altitudinal gradient from subalpine to mid‐alpine vegetation. One possible explanation for the delayed flowering might be that the early flowering subalpine populations have gradually been lost and we only have high‐altitude populations left. The species with the greatest advance in flowering, albeit with large variation, was *Arabidopsis suecica*. This is a postglacial allopolyploid species formed via hybridization of *A. thaliana* and *A. arenosa* (Burns et al., 2021), which was first recorded in Central Norway in 1939. While all the other assessed taxa are native, this is a quickly expanding non‐native species (Elven et al., 2018), and non‐native species may be more plastic in phenology than natives (Zettlemoyer et al., 2019).

### 4.2 Abundance and diversity

The abundance of both limnic zooplankton and boreal forest breeding birds decreased over time, and in relation to temperature increases. The decrease in boreal bird abundances was consistent in direction with findings from other boreal ecosystems, for example in Finland where bird densities decreased by 10% (Virkkala et al., 2018), as well as global syntheses emphasizing the negative effect of temperature on population size (Spooner et al., 2018). Analysis of long‐term zooplankton time series suggests that increased climate variability may increase the frequency of extreme demographic events either increasing or decreasing long‐run population growth (Drake, 2005). Simulations highly support this view, by showing that the amplitude of fluctuations of the herbivorous zooplankton stock increases with temperature while the mean biomass and minimum values decrease in comparison with steady‐state predictions (Norberg & DeAngelis, 1997). Zooplankton abundance trends may also have been influenced by the establishment of a regionally non‐native species, *Mysis relicta* in 1979 (Koksvik et al., 2009).

The species richness of mayfly and stonefly larvae in upland rivers was largely stable over time and temperature. This finding should be interpreted with caution, due to the change in sampling methodology within the time frame of the study. Although the estimated change in species richness with temperature was not significantly different from zero, its direction (species gain with warming) is consistent with elevational advances in the distribution of low‐elevation stoneflies in the Appalachians, with increases in elevation of up to 250 m for 0.7°C warming (Sheldon, 2012).

### 4.3 Distributions

We found generally no change in species distributions (latitude) over time, or in relation to temperature. This is in contrast to multiple studies both marine (Hastings et al., 2020) and terrestrial with a meta‐analysis finding an average poleward shift of 17 km per decade (Chen et al., 2011). However, unlike broader scale analyses of distribution shifts related to changing climate, our approach did not concentrate on distribution edges (we used the 90th quantile latitude within our study area of Central Norway, to avoid issues with temporal bias in the natural history collections). Caution has been advised when using museum records to infer distribution shifts, mainly due to biases in sampling, which were also likely present in the occurrence datasets we used (Przeslawski et al., 2012). For some terrestrial taxa, we actually found some signs of decreasing latitude, with decreasing latitude of animal distributions over time, and plant distributions with temperature. Lenoir et al. (2020) also found relatively stable latitudinal distributions, with no clear signs of range shift at the mean trailing edge (mean velocity: −0.17 ± 1.61 km year^−1^), centroid (2.41 ± 2.45 km year^−1^), or leading edge (0.81 ± 0.65 km year^−1^). The only terrestrial taxonomic groups with significant range shift in their meta‐analysis were reptiles (with an equatorial shift in trailing edge), arachnids (poleward moving leading edge), and insects (poleward moving trailing edge and centroid). Their suggested mechanisms for distribution stability are antagonistic effects between climatic effects and human‐related drivers, such as habitat loss and fragmentation. It may also be the case, that for our regional‐scale study, latitudinal distributions do not occur at a suitable scale for testing climate signals, potentially underestimating the effect of warming (VanDerWal et al., 2013), and in a topographically heterogenous region, such as ours, elevational distribution shifts may be more closely related to climate. However, elevational distributions can also have close associations with land‐use changes (Guo et al., 2018), again complicating the overall dynamics.

### 4.4 Synthesis

Contrary to our hypothesis 2, the effect size of ecological responses to both time and temperature did not increase with the duration of the dataset. In fact, the opposite was the case, with the effect sizes decreasing with dataset duration. For the temporal trends, this was not due to differences in duration between different ecological responses, since the same pattern was found within both distribution and phenological responses alone. However, for temperature, there was no trend in effect size magnitude against study duration within either the distribution or phenology response types. This contrast may be explained by the shorter duration studies only overlapping with the latter period of warming (Figure 2), while the longer studies include the period of climatic stability (or slight cooling) between 1946 and 1979, and in the case of the longest duration studies, these span a period for which we do not have measured temperature data (before 1900). This highlights the importance of natural history collections as long‐term ecological repositories, for understanding the dynamics of ecological change over relevant decadal to centurial timescales (Meineke et al., 2019). Indeed, the natural history collections predate an accurate temperature record for the region.

Our hypothesis 3 that thresholds in ecological response temporal trends would occur at the same time as thresholds in temperature temporal trends was also not supported. Breakpoints were found to be closer to (but still different from) a normal distribution than a bimodal distribution with peaks in the years with breakpoints in the temperature trends. This may be as ecological responses are lagged responses to temperature (Menéndez et al., 2006; Walther et al., 2002) and lend support to the premise that ecological systems are often in disequilibrium with climate (Svenning & Sandel, 2013).

While climate change can have intense impacts on ecological systems, it is not the sole element of global environmental change. Other factors are also changing simultaneously, such as land use (and water/ocean use), pollution, and the spread of non‐native species (Steffen et al., 2015; Vitousek, 1994). Land‐use and land‐cover changes can impact upon species distributions. In our study region, land cover, and in particular urbanization and infrastructure development, is an important determinant of species distribution and community composition (Petersen et al., 2020). In addition, changes in large‐herbivore densities (through livestock management and directed hunting of wild ungulates) within our study region have been dramatic (Speed et al., 2019), and these have been shown to affect the distribution of plant and fungi species (Speed et al., 2020). In forest ecosystems, the presence of old‐growth stands has been shown to buffer bird populations from the effects of warming (Betts et al., 2018). Aquatic invertebrates are particularly susceptible to a range of land uses, as well as pollution and water‐course management (Collier et al., 2016). Recovery from the impacts of acid precipitation may also mask some of the effects of climate change on freshwater ecological responses (Warren et al., 2017). Thus, other elements of global environmental change can also have effects on the ecological responses assessed here and may contribute to explaining the findings that deviated from our hypotheses.

Our study is based on natural history collections. As such the estimates of ecological responses should be independent of potential publication biases. However, natural history collections themselves are not without bias. Well‐known biases exist in natural history collections, within dimensions including space, time, taxonomy, environment, and species traits (Meineke & Daru, 2021; Speed et al., 2018). In our study the phenological and distribution datasets may be particularly susceptible to biases as these are not quantified based on designed or consistent collection patterns. However, there are steps that can be taken to assess and account for biases (Meineke & Daru, 2021), and we have endeavored to do so in our analyses, for example by assessing peak flowering rather than earliest flowering. Perhaps more pertinent is that the ecological responses analyzed here were a selection of potential ecological responses that could have been quantified from our natural history collections. This selection was based on the interests and understanding of the study's authors, and as such is not objective, and biases in study selection may have occurred.

In this study we used natural history collections spanning over 250 years, to quantify a range of ecological responses, including phenology, abundance, diversity, and distributions over time and with temperature within the region of Central Norway. By combining analyses across ecological variables and taxa we demonstrate how climate change can form a footprint on ecological systems at a regional scale. We identified aligned trends in ecological responses over time and temperature, with decreasing abundances of zooplankton and breeding birds and earlier plant flowering phenology but largely regionally stable distributions and diversity. Investigation of climate fingerprints at such timescales and as regionally specific as we have achieved here is rare. We contend that natural history collections are the sole window on such a broad spectrum of ecological responses at this timescale and that natural history collections are an essential source for ecological research.

## AUTHOR CONTRIBUTIONS

**James D. M. Speed:** Conceptualization (lead); formal analysis (lead); investigation (lead); visualization (lead); writing – original draft (lead). **Ann Evankow:** Formal analysis (supporting); investigation (supporting); writing – review and editing (supporting). **Tanja Kofod Petersen:** Conceptualization (supporting); formal analysis (supporting); investigation (supporting); writing – review and editing (equal). **Peter Ranke:** Investigation (equal); writing – review and editing (equal). **Nellie Nilsen:** Investigation (equal); writing – review and editing (equal). **Grace Turner:** Investigation (equal); writing – review and editing (equal). **Kaare Aagaard:** Investigation (equal); writing – review and editing (equal). **Torkild Bakken:** Conceptualization (equal); methodology (equal); writing – review and editing (equal). **Jan G. Davidsen:** Conceptualization (equal); investigation (equal); writing – review and editing (equal). **Glenn Dunshea:** Conceptualization (equal); investigation (equal); writing – review and editing (equal). **Anders G. Finstad:** Conceptualization (equal); investigation (equal); writing – review and editing (equal). **Kristian Hassel:** Conceptualization (equal); investigation (equal); writing – review and editing (equal). **Magne Husby:** Conceptualization (equal); investigation (equal); writing – review and editing (equal). **Karstein Hårsaker:** Conceptualization (equal); investigation (equal); writing – review and editing (equal). **Jan Ivar Koksvik:** Investigation (equal); writing – review and editing (equal). **Tommy Prestø:** Conceptualization (equal); investigation (equal); writing – review and editing (equal). **Vibekke Vange:** Conceptualization (equal); investigation (equal); writing – review and editing (equal).

## Supporting information


Figure S1 & S2

Table S1

Table S2

## Data Availability

All source data and derived data (i.e. estimates of flowering from the herbarium specimens) for this study are publicly available (DOIs/citations are given in Table 1 and in text).
