# Supplementary material for: A regionally coherent ecological fingerprint of climate change, evidenced from natural history collections
Source: Ecol Evol. 2022 Nov 1;12(11):e9471. doi: 10.1002/ece3.9471 (PMC9627063; doi:10.1002/ece3.9471)
Supplement: Supplementary file 1 — Figure S1 & S2 [file ECE3-12-e9471-s003.docx]

## Supporting Information


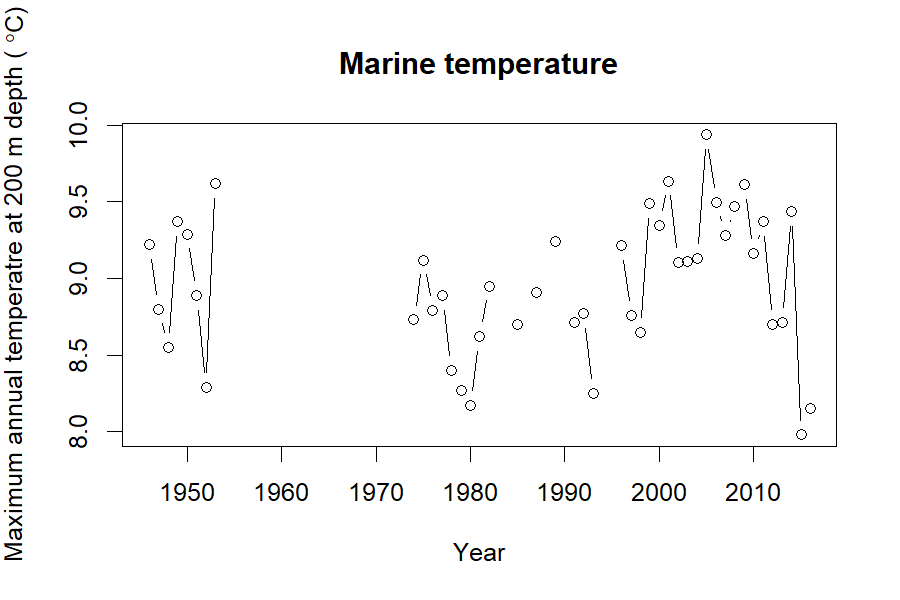


Figure S1. Marine temperature data presented as the annual maximum record at the sampling station Bud, 200 m depth.


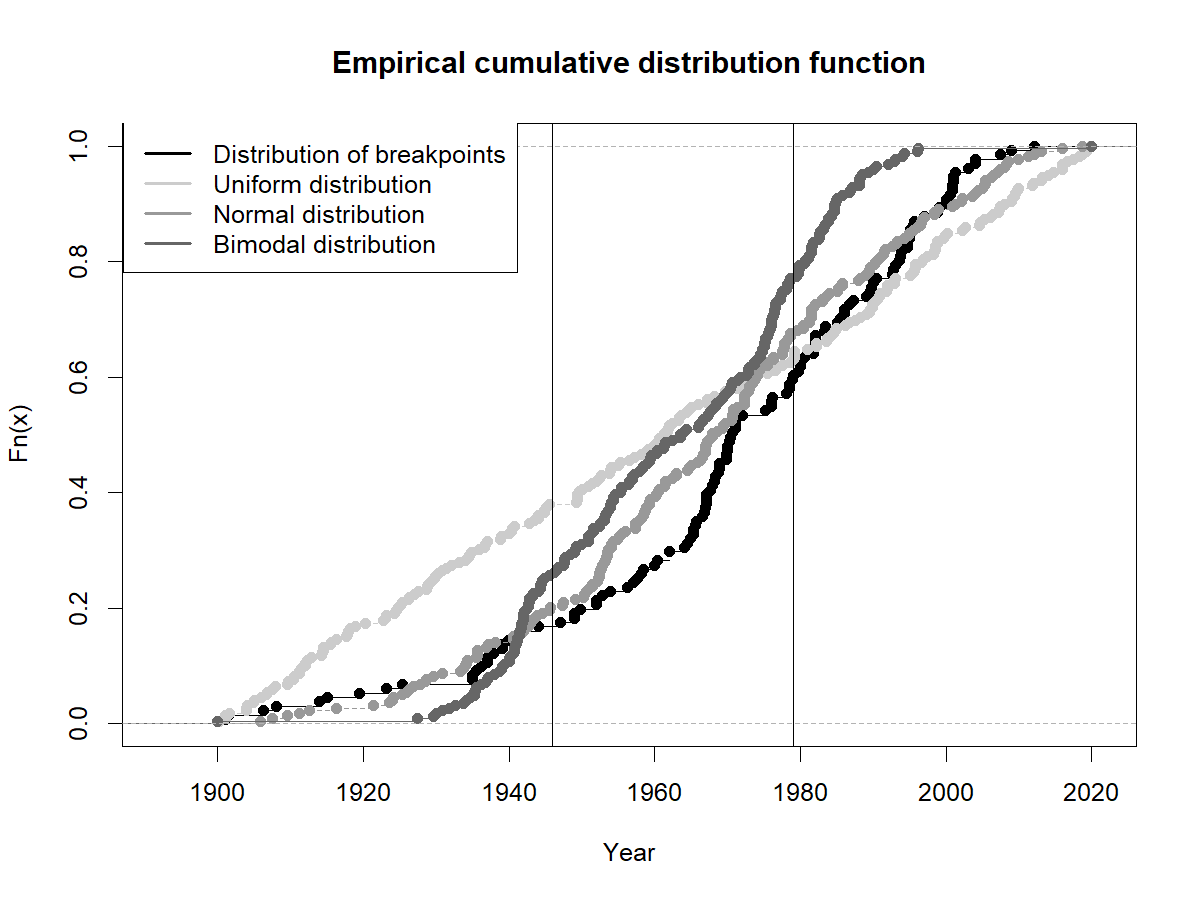


Figure S2. Cumulative distribution functions for the years of significant breakpoints within linear regression models of species distributions and plant phenology (black). Simulated distributions are shown in grey, based on uniform, normal (truncated between 1900 and 2020 with a mean and standard deviation from the observed distributions) and bimodal (with two means at 1946 and 1979 and standard deviation as 0.25 observed value).
