# Supplementary material for: A regionally coherent ecological fingerprint of climate change, evidenced from natural history collections
Source: Ecol Evol. 2022 Nov 1;12(11):e9471. doi: 10.1002/ece3.9471 (PMC9627063; doi:10.1002/ece3.9471)
Supplement: Supplementary file 3 — Table S2 [file ECE3-12-e9471-s001.docx]

**Supporting Table S2** Regression slopes and standard errors for individual species distribution leading edge (latitude, 90^th^ percentile) against year and temperature. Species, kingdom and phylum are shown along with the number of years with data (N) and the duration between the earliest and latest years.

| **Species** | **Kingdom** | **Phylum** | **N** | **Duration** | **Year**  **Slope** | **SE** | **Temperature**  **Slope** | **SE** |
| --- | --- | --- | --- | --- | --- | --- | --- | --- |
| *Acanthis flammea* | Animalia | Chordata | 9 | 99 | -0.01605 | 0.00136 | -0.31408 | 0.1199 |
| *Accipiter gentilis* | Animalia | Chordata | 18 | 56 | 0.00176 | 0.00644 | -0.00903 | 0.0918 |
| *Accipiter nisus* | Animalia | Chordata | 17 | 114 | -0.00213 | 0.00372 | -0.11204 | 0.08476 |
| *Achillea ptarmica* | Plantae | Tracheophyta | 30 | 49 | -0.01845 | 0.00914 | -0.30668 | 0.11211 |
| *Achnatherum calamagrostis* | Plantae | Tracheophyta | 78 | 118 | 0.00593 | 0.00278 | -0.00036 | 0.08066 |
| *Aconitum nasutum* | Plantae | Tracheophyta | 15 | 91 | 0.00397 | 0.00487 | 0.07095 | 0.13651 |
| *Actaea spicata* | Plantae | Tracheophyta | 39 | 61 | 0.00137 | 0.00527 | -0.13172 | 0.10107 |
| *Aegolius funereus* | Animalia | Chordata | 9 | 105 | -0.00043 | 0.00545 | -0.08615 | 0.17723 |
| *Agrostis canina* | Plantae | Tracheophyta | 23 | 54 | 0.01225 | 0.00881 | 0.1109 | 0.19496 |
| *Agrostis capillaris* | Plantae | Tracheophyta | 35 | 54 | -0.01759 | 0.00846 | -0.39627 | 0.12572 |
| *Agrostis mertensii* | Plantae | Tracheophyta | 29 | 63 | 0.00242 | 0.00876 | -0.07733 | 0.19133 |
| *Agrostis stolonifera* | Plantae | Tracheophyta | 25 | 54 | 0.00093 | 0.00792 | -0.15195 | 0.13755 |
| *Agrypnia obsoleta* | Animalia | Arthropoda | 19 | 70 | -0.00115 | 0.00442 | -0.01409 | 0.08977 |
| *Alchemilla alpina* | Plantae | Tracheophyta | 32 | 57 | -0.03068 | 0.00811 | -0.47751 | 0.14679 |
| *Alchemilla filicaulis* | Plantae | Tracheophyta | 4 | 63 | 0.02402 | 0.00996 | 0.10008 | 1.18943 |
| *Alchemilla glabra* | Plantae | Tracheophyta | 26 | 74 | 0.00123 | 0.00496 | -0.08531 | 0.11476 |
| *Alchemilla glomerulans* | Plantae | Tracheophyta | 17 | 59 | 0.0034 | 0.0166 | 0.03693 | 0.36412 |
| *Alchemilla monticola* | Plantae | Tracheophyta | 23 | 73 | 0.00843 | 0.0033 | 0.17128 | 0.0861 |
| *Alchemilla murbeckiana* | Plantae | Tracheophyta | 9 | 75 | -0.00054 | 0.00347 | 0.02856 | 0.0627 |
| *Alchemilla norvegica* | Plantae | Tracheophyta | 8 | 70 | 0.01373 | 0.01024 | -0.03211 | 0.30342 |
| *Alchemilla subcrenata* | Plantae | Tracheophyta | 8 | 63 | 0.00659 | 0.00473 | 0.1108 | 0.14888 |
| *Alchemilla wichurae* | Plantae | Tracheophyta | 21 | 60 | -0.00941 | 0.0096 | 0.0366 | 0.17604 |
| *Alisma plantago-aquatica* | Plantae | Tracheophyta | 4 | 67 | 0.00202 | 0.0073 | 0.03195 | 0.15859 |
| *Alle alle* | Animalia | Chordata | 4 | 51 | -0.00529 | 0.01206 | -0.51236 | 0.49413 |
| *Allium oleraceum* | Plantae | Tracheophyta | 20 | 67 | 0.00654 | 0.00519 | 0.0558 | 0.10696 |
| *Alnus glutinosa* | Plantae | Tracheophyta | 4 | 51 | -0.00059 | 0.01201 | 1.86002 | 0.85037 |
| *Alopecurus geniculatus* | Plantae | Tracheophyta | 28 | 53 | -0.00973 | 0.0068 | -0.33248 | 0.11468 |
| *Ampullaceana balthica* | Animalia | Mollusca | 19 | 49 | 0.01144 | 0.00947 | 0.19983 | 0.11666 |
| *Anabolia brevipennis* | Animalia | Arthropoda | 2 | 53 | 0.00021 | NA | 0.00586 | NA |
| *Anas platyrhynchos* | Animalia | Chordata | 2 | 83 | -0.02421 | NA | -1.48193 | NA |
| *Andromeda polifolia* | Plantae | Tracheophyta | 28 | 104 | -0.01238 | 0.00653 | -0.24138 | 0.13945 |
| *Anemone nemorosa* | Plantae | Tracheophyta | 34 | 67 | 0.00061 | 0.007 | -0.20339 | 0.12226 |
| *Antennaria alpina* | Plantae | Tracheophyta | 16 | 56 | -0.01338 | 0.01042 | -0.22554 | 0.23057 |
| *Antennaria dioica* | Plantae | Tracheophyta | 42 | 77 | -0.01178 | 0.00555 | -0.30138 | 0.12632 |
| *Anthelia juratzkana* | Plantae | Marchantiophyta | 3 | 68 | 0.01598 | 0.00381 | 0.27642 | 0.1623 |
| *Anthoxanthum nipponicum* | Plantae | Tracheophyta | 5 | 53 | -0.00204 | 0.025 | 0.21103 | 0.59807 |
| *Anthoxanthum nitens* | Plantae | Tracheophyta | 83 | 120 | 0.0029 | 0.00291 | -0.05694 | 0.07794 |
| *Anthoxanthum odoratum* | Plantae | Tracheophyta | 31 | 104 | -0.017 | 0.0065 | -0.3615 | 0.12059 |
| *Anthyllis vulneraria* | Plantae | Tracheophyta | 29 | 69 | 0.00655 | 0.00565 | -0.08706 | 0.11157 |
| *Antitrichia curtipendula* | Plantae | Bryophyta | 9 | 57 | 0.0161 | 0.00657 | 0.20124 | 0.27175 |
| *Apatania stigmatella* | Animalia | Arthropoda | 21 | 64 | -0.00845 | 0.00988 | 0.1498 | 0.14357 |
| *Apatania wallengreni* | Animalia | Arthropoda | 11 | 55 | 0.00075 | 0.00035 | 0.01135 | 0.01165 |
| *Apocatops nigrita* | Animalia | Arthropoda | 4 | 62 | -0.01187 | 0.01509 | 2.5657 | 0.40623 |
| *Aquila chrysaetos* | Animalia | Chordata | 16 | 57 | -0.02291 | 0.01463 | -0.38615 | 0.21316 |
| *Arabidopsis arenosa* | Plantae | Tracheophyta | 8 | 54 | 0.00416 | 0.00896 | 0.00371 | 0.2203 |
| *Arabidopsis suecica* | Plantae | Tracheophyta | 7 | 60 | 0.00831 | 0.0063 | 0.18297 | 0.15406 |
| *Arabidopsis thaliana* | Plantae | Tracheophyta | 20 | 84 | -0.00259 | 0.00356 | -0.09316 | 0.07692 |
| *Arabis alpina* | Plantae | Tracheophyta | 24 | 65 | -0.01794 | 0.0119 | -0.27535 | 0.23126 |
| *Arabis hirsuta* | Plantae | Tracheophyta | 27 | 65 | 0.00278 | 0.00564 | -0.0691 | 0.12396 |
| *Arctostaphylos alpinus* | Plantae | Tracheophyta | 75 | 119 | 0.00531 | 0.00318 | -0.025 | 0.08965 |
| *Ardea cinerea* | Animalia | Chordata | 4 | 59 | -0.01245 | 0.00249 | -0.26738 | 0.24324 |
| *Arenaria interpres* | Animalia | Chordata | 2 | 82 | -0.00328 | NA | -0.19112 | NA |
| *Arenaria serpyllifolia* | Plantae | Tracheophyta | 14 | 61 | 0.01743 | 0.00776 | 0.24951 | 0.18957 |
| *Armiger crista* | Animalia | Mollusca | 8 | 90 | -0.00756 | 0.00092 | -0.14965 | 0.05996 |
| *Arrhenatherum elatius* | Plantae | Tracheophyta | 17 | 59 | 0.01748 | 0.00859 | 0.12108 | 0.19049 |
| *Artemisia norvegica* | Plantae | Tracheophyta | 10 | 59 | -0.00132 | 0.00156 | -0.00999 | 0.03366 |
| *Artemisia vulgaris* | Plantae | Tracheophyta | 16 | 70 | -0.00069 | 0.00504 | -0.17633 | 0.09929 |
| *Asplenium septentrionale* | Plantae | Tracheophyta | 10 | 52 | -0.00396 | 0.0045 | -0.1338 | 0.06046 |
| *Asplenium trichomanes* | Plantae | Tracheophyta | 23 | 84 | -0.00699 | 0.00633 | -0.3642 | 0.12794 |
| *Asplenium viride* | Plantae | Tracheophyta | 35 | 78 | -0.00123 | 0.00492 | -0.14536 | 0.10308 |
| *Astragalus alpinus* | Plantae | Tracheophyta | 32 | 66 | -0.00408 | 0.00798 | -0.10267 | 0.16851 |
| *Astragalus frigidus* | Plantae | Tracheophyta | 14 | 54 | -0.00739 | 0.00206 | -0.09445 | 0.03788 |
| *Astragalus norvegicus* | Plantae | Tracheophyta | 18 | 59 | 0.00197 | 0.00924 | -0.09492 | 0.15268 |
| *Atheta laevicauda* | Animalia | Arthropoda | 5 | 69 | -0.03312 | 0.00123 | -0.76603 | 0.46462 |
| *Atheta lativentris* | Animalia | Arthropoda | 2 | 61 | -0.00452 | NA | 1.10463 | NA |
| *Athripsodes aterrimus* | Animalia | Arthropoda | 11 | 70 | -0.00327 | 0.00222 | -0.00318 | 0.06086 |
| *Athripsodes cinereus* | Animalia | Arthropoda | 7 | 69 | 0.00171 | 0.0051 | 0.10798 | 0.10926 |
| *Athyrium filix-femina* | Plantae | Tracheophyta | 34 | 67 | -0.00518 | 0.00661 | -0.31742 | 0.12429 |
| *Atocion rupestre* | Plantae | Tracheophyta | 17 | 55 | -0.02522 | 0.00631 | -0.25943 | 0.16984 |
| *Atriplex glabriuscula* | Plantae | Tracheophyta | 4 | 54 | 0.01131 | 0.01775 | -0.61164 | 0.70014 |
| *Atriplex prostrata* | Plantae | Tracheophyta | 10 | 49 | -0.00998 | 0.01597 | -0.30206 | 0.24422 |
| *Aulacomnium palustre* | Plantae | Bryophyta | 3 | 73 | 0.02067 | 0.00946 | -0.8506 | 2.40471 |
| *Aulacomnium turgidum* | Plantae | Bryophyta | 3 | 107 | 0.00198 | 0.0058 | 0.00107 | 0.17878 |
| *Avenella flexuosa* | Plantae | Tracheophyta | 34 | 54 | -0.03075 | 0.00875 | -0.40274 | 0.1542 |
| *Avenula pubescens* | Plantae | Tracheophyta | 31 | 69 | 0.0097 | 0.00614 | -0.11667 | 0.14565 |
| *Baetis rhodani* | Animalia | Arthropoda | 47 | 49 | -0.00838 | 0.00622 | 0.0029 | 0.08628 |
| *Balanus balanus* | Animalia | Arthropoda | 10 | 71 | 0.00029 | 0.00539 | -0.19834 | 0.19806 |
| *Balanus crenatus* | Animalia | Arthropoda | 7 | 51 | -0.00891 | 0.00347 | -0.04254 | 0.11857 |
| *Barbarea stricta* | Plantae | Tracheophyta | 24 | 56 | 0.00968 | 0.00669 | 0.12125 | 0.12074 |
| *Barbarea vulgaris* | Plantae | Tracheophyta | 14 | 54 | 0.0134 | 0.00749 | -0.11934 | 0.17909 |
| *Barbilophozia barbata* | Plantae | Marchantiophyta | 3 | 65 | 0.0034 | 0.006 | -0.2066 | 0.35878 |
| *Barbilophozia sudetica* | Plantae | Marchantiophyta | 2 | 62 | 0.04146 | NA | 2.69182 | NA |
| *Bartramia pomiformis* | Plantae | Bryophyta | 3 | 72 | 0.01678 | 0.00396 | 1.16218 | 0.19537 |
| *Bartsia alpina* | Plantae | Tracheophyta | 38 | 112 | -0.01908 | 0.00541 | -0.26063 | 0.13052 |
| *Bathyomphalus contortus* | Animalia | Mollusca | 16 | 91 | -0.00492 | 0.00272 | -0.0114 | 0.0839 |
| *Betula nana* | Plantae | Tracheophyta | 33 | 105 | -0.01121 | 0.0064 | -0.31712 | 0.13625 |
| *Betula pubescens* | Plantae | Tracheophyta | 35 | 104 | -0.00529 | 0.00582 | -0.27893 | 0.11071 |
| *Blepharostoma trichophyllum* | Plantae | Marchantiophyta | 27 | 112 | 0.02277 | 0.0049 | 0.46102 | 0.15408 |
| *Blysmus rufus* | Plantae | Tracheophyta | 59 | 102 | -0.00031 | 0.0028 | -0.13525 | 0.07641 |
| *Bolyphantes alticeps* | Animalia | Arthropoda | 2 | 56 | -0.00058 | NA | -0.03787 | NA |
| *Bombus pascuorum* | Animalia | Arthropoda | 3 | 63 | -0.01659 | 0.00298 | -0.19581 | 0.27524 |
| *Bombus pratorum* | Animalia | Arthropoda | 2 | 63 | -0.02214 | NA | -0.46619 | NA |
| *Botrychium lunaria* | Plantae | Tracheophyta | 41 | 69 | 0.0028 | 0.00519 | -0.21016 | 0.10718 |
| *Botryobasidium vagum* | Fungi | Basidiomycota | 7 | 61 | 0.00114 | 0.01474 | -0.10195 | 0.46759 |
| *Brachythecium glareosum* | Plantae | Bryophyta | 5 | 108 | 0.01028 | 0.00903 | 0.39706 | 0.24057 |
| *Brachythecium rivulare* | Plantae | Bryophyta | 3 | 65 | 0.0062 | 0.00363 | 0.85036 | 0.37604 |
| *Brachythecium rutabulum* | Plantae | Bryophyta | 3 | 65 | 0.0037 | 0.00193 | -0.70261 | 1.57958 |
| *Brachythecium salebrosum* | Plantae | Bryophyta | 8 | 95 | 0.00866 | 0.00576 | 0.15972 | 0.25131 |
| *Bryum pallens* | Plantae | Bryophyta | 9 | 109 | 0.01171 | 0.00608 | 0.29852 | 0.17224 |
| *Bryum pseudotriquetrum* | Plantae | Bryophyta | 11 | 106 | 0.01151 | 0.00631 | 0.24426 | 0.20194 |
| *Buccinum undatum* | Animalia | Mollusca | 25 | 64 | 0.00014 | 0.00306 | 0.04715 | 0.07555 |
| *Caenis horaria* | Animalia | Arthropoda | 20 | 58 | -0.00141 | 0.00449 | -0.05406 | 0.07288 |
| *Calamagrostis purpurea* | Plantae | Tracheophyta | 42 | 69 | -0.0093 | 0.00618 | -0.25032 | 0.12286 |
| *Calamagrostis stricta* | Plantae | Tracheophyta | 17 | 60 | 0.00593 | 0.01546 | -0.2983 | 0.22547 |
| *Callicorixa wollastoni* | Animalia | Arthropoda | 13 | 50 | 0.0134 | 0.01532 | 0.3037 | 0.20367 |
| *Calliergonella lindbergii* | Plantae | Bryophyta | 3 | 69 | 0.00512 | 0.00692 | 0.20425 | 0.18482 |
| *Callitriche brutia* | Plantae | Tracheophyta | 49 | 99 | -0.00197 | 0.00442 | 0.09367 | 0.11696 |
| *Callitriche palustris* | Plantae | Tracheophyta | 9 | 55 | -0.0036 | 0.01454 | -0.1056 | 0.20961 |
| *Campanula latifolia* | Plantae | Tracheophyta | 24 | 52 | -0.00386 | 0.00871 | -0.108 | 0.15148 |
| *Campanula rotundifolia* | Plantae | Tracheophyta | 34 | 49 | -0.03612 | 0.00831 | -0.49893 | 0.1249 |
| *Campylium protensum* | Plantae | Bryophyta | 8 | 113 | 0.01369 | 0.00336 | 0.33414 | 0.12053 |
| *Campylium stellatum* | Plantae | Bryophyta | 17 | 99 | 0.01419 | 0.00785 | 0.46708 | 0.2509 |
| *Cardamine amara* | Plantae | Tracheophyta | 26 | 55 | -0.00248 | 0.00527 | 0.0751 | 0.12554 |
| *Cardamine bellidifolia* | Plantae | Tracheophyta | 8 | 61 | -0.00821 | 0.01963 | 0.08623 | 0.43156 |
| *Cardamine flexuosa* | Plantae | Tracheophyta | 23 | 76 | -0.0035 | 0.0039 | -0.14175 | 0.07741 |
| *Cardamine pratensis* | Plantae | Tracheophyta | 27 | 50 | 0.00657 | 0.00878 | -0.21994 | 0.12472 |
| *Carex acuta* | Plantae | Tracheophyta | 7 | 70 | 0.00257 | 0.00762 | -0.02393 | 0.14602 |
| *Carex adelostoma* | Plantae | Tracheophyta | 39 | 58 | 0.00963 | 0.00765 | 0.08174 | 0.15103 |
| *Carex appropinquata* | Plantae | Tracheophyta | 7 | 53 | 0.01638 | 0.01768 | -0.16144 | 0.50532 |
| *Carex atrata* | Plantae | Tracheophyta | 42 | 65 | -0.00154 | 0.00742 | -0.34144 | 0.14771 |
| *Carex atrofusca* | Plantae | Tracheophyta | 33 | 63 | -0.00666 | 0.01028 | -0.12154 | 0.18459 |
| *Carex bigelowii* | Plantae | Tracheophyta | 44 | 119 | -0.00462 | 0.00624 | -0.25925 | 0.1416 |
| *Carex brunnescens* | Plantae | Tracheophyta | 35 | 69 | 0.00538 | 0.00784 | -0.15382 | 0.17551 |
| *Carex buxbaumii* | Plantae | Tracheophyta | 34 | 62 | 0.01253 | 0.00607 | -0.07152 | 0.11797 |
| *Carex canescens* | Plantae | Tracheophyta | 41 | 65 | 0.00283 | 0.00531 | -0.10568 | 0.11976 |
| *Carex capillaris* | Plantae | Tracheophyta | 46 | 78 | -0.00086 | 0.00464 | -0.05734 | 0.1007 |
| *Carex chordorrhiza* | Plantae | Tracheophyta | 27 | 57 | 0.00272 | 0.00936 | -0.00795 | 0.16302 |
| *Carex dacica* | Plantae | Tracheophyta | 41 | 120 | 0.00366 | 0.00361 | 0.16951 | 0.11401 |
| *Carex demissa* | Plantae | Tracheophyta | 44 | 84 | 0.00235 | 0.00429 | -0.15455 | 0.09137 |
| *Carex digitata* | Plantae | Tracheophyta | 40 | 64 | -0.00041 | 0.0048 | -0.11108 | 0.1001 |
| *Carex dioica* | Plantae | Tracheophyta | 47 | 101 | 0.00596 | 0.00432 | -0.10813 | 0.11157 |
| *Carex echinata* | Plantae | Tracheophyta | 32 | 55 | -0.01638 | 0.00805 | -0.07216 | 0.14545 |
| *Carex flacca* | Plantae | Tracheophyta | 18 | 69 | 0.00932 | 0.00791 | 0.05539 | 0.17517 |
| *Carex flava* | Plantae | Tracheophyta | 61 | 119 | 0.00127 | 0.00305 | -0.13481 | 0.06984 |
| *Carex fuliginosa* | Plantae | Tracheophyta | 8 | 59 | -0.00396 | 0.00195 | -0.07909 | 0.04177 |
| *Carex heleonastes* | Plantae | Tracheophyta | 3 | 63 | -0.00011 | 0.00106 | 0.06316 | 0.04522 |
| *Carex hostiana* | Plantae | Tracheophyta | 36 | 85 | 0.0054 | 0.00481 | 0.02033 | 0.10537 |
| *Carex lachenalii* | Plantae | Tracheophyta | 26 | 63 | -0.0102 | 0.00851 | -0.32217 | 0.16495 |
| *Carex lasiocarpa* | Plantae | Tracheophyta | 28 | 63 | 0.00443 | 0.00679 | 0.05391 | 0.13332 |
| *Carex lepidocarpa* | Plantae | Tracheophyta | 25 | 85 | -0.00543 | 0.00438 | -0.19331 | 0.07874 |
| *Carex leporina* | Plantae | Tracheophyta | 22 | 70 | -0.00578 | 0.00738 | -0.16524 | 0.1379 |
| *Carex limosa* | Plantae | Tracheophyta | 40 | 77 | 0.00893 | 0.0061 | 0.01414 | 0.13206 |
| *Carex maritima* | Plantae | Tracheophyta | 14 | 65 | 0.02111 | 0.00777 | 0.21396 | 0.23571 |
| *Carex microglochin* | Plantae | Tracheophyta | 24 | 59 | 0.00895 | 0.0086 | -0.0803 | 0.13669 |
| *Carex myosuroides* | Plantae | Tracheophyta | 10 | 63 | -0.00574 | 0.01273 | -0.4201 | 0.26764 |
| *Carex nigra* | Plantae | Tracheophyta | 62 | 106 | 0.00518 | 0.00363 | -0.06595 | 0.09643 |
| *Carex norvegica* | Plantae | Tracheophyta | 38 | 71 | 0.00785 | 0.00763 | -0.1922 | 0.16632 |
| *Carex oederi* | Plantae | Tracheophyta | 77 | 105 | 0.00429 | 0.00292 | 0.06344 | 0.07664 |
| *Carex ornithopoda* | Plantae | Tracheophyta | 30 | 64 | -0.00984 | 0.00542 | -0.22615 | 0.10379 |
| *Carex pallescens* | Plantae | Tracheophyta | 31 | 65 | -0.00147 | 0.00609 | -0.27148 | 0.11133 |
| *Carex panicea* | Plantae | Tracheophyta | 45 | 119 | -0.00445 | 0.00393 | -0.20155 | 0.09743 |
| *Carex pauciflora* | Plantae | Tracheophyta | 28 | 51 | -0.02747 | 0.01032 | -0.10948 | 0.17485 |
| *Carex pilulifera* | Plantae | Tracheophyta | 35 | 68 | 0.00074 | 0.00388 | -0.13071 | 0.0851 |
| *Carex pulicaris* | Plantae | Tracheophyta | 37 | 66 | 0.02329 | 0.00458 | 0.00298 | 0.13573 |
| *Carex rariflora* | Plantae | Tracheophyta | 25 | 63 | 0.00684 | 0.01023 | 0.135 | 0.19597 |
| *Carex rostrata* | Plantae | Tracheophyta | 51 | 77 | 0.00227 | 0.00511 | -0.0478 | 0.11677 |
| *Carex rotundata* | Plantae | Tracheophyta | 16 | 55 | 0.02197 | 0.0169 | -0.09169 | 0.26738 |
| *Carex rufina* | Plantae | Tracheophyta | 8 | 63 | 0.02527 | 0.01769 | -0.25125 | 0.3952 |
| *Carex rupestris* | Plantae | Tracheophyta | 33 | 74 | -0.0049 | 0.00784 | -0.29039 | 0.14495 |
| *Carex saxatilis* | Plantae | Tracheophyta | 37 | 82 | -0.00585 | 0.0083 | -0.14103 | 0.20168 |
| *Carex simpliciuscula* | Plantae | Tracheophyta | 29 | 63 | 0.0068 | 0.00632 | -0.08937 | 0.12283 |
| *Carex vaginata* | Plantae | Tracheophyta | 38 | 65 | -0.00548 | 0.00776 | -0.27737 | 0.17728 |
| *Carex vesicaria* | Plantae | Tracheophyta | 24 | 62 | -0.00019 | 0.00559 | -0.15482 | 0.09765 |
| *Carum carvi* | Plantae | Tracheophyta | 23 | 55 | -0.0099 | 0.00891 | -0.35518 | 0.10083 |
| *Catoscopium nigritum* | Plantae | Bryophyta | 3 | 61 | 0.016 | 0.00791 | 0.64421 | 0.31052 |
| *Centaurea cyanus* | Plantae | Tracheophyta | 33 | 96 | 0.00204 | 0.00177 | -0.07037 | 0.04766 |
| *Centroptilum luteolum* | Animalia | Arthropoda | 32 | 57 | -0.01073 | 0.00621 | -0.03059 | 0.08971 |
| *Cephalozia bicuspidata* | Plantae | Marchantiophyta | 13 | 106 | 0.01285 | 0.0029 | 0.38388 | 0.10614 |
| *Cepphus grylle* | Animalia | Chordata | 6 | 55 | -0.03247 | 0.00747 | -0.80364 | 0.48702 |
| *Cerastium alpinum* | Plantae | Tracheophyta | 47 | 80 | -0.0131 | 0.00566 | -0.39208 | 0.13578 |
| *Cerastium cerastoides* | Plantae | Tracheophyta | 24 | 60 | -0.02885 | 0.01066 | -0.20772 | 0.26162 |
| *Cerastium fontanum* | Plantae | Tracheophyta | 35 | 55 | -0.0023 | 0.00868 | -0.34707 | 0.13686 |
| *Cerastium glomeratum* | Plantae | Tracheophyta | 10 | 74 | 0.00939 | 0.0064 | 0.18379 | 0.33448 |
| *Cerastium holosteoides* | Plantae | Tracheophyta | 72 | 117 | -0.00014 | 0.0024 | -0.04795 | 0.07225 |
| *Ceratopsyche newae* | Animalia | Arthropoda | 34 | 67 | -0.00981 | 0.00483 | -0.04043 | 0.07258 |
| *Chenopodium album* | Plantae | Tracheophyta | 16 | 74 | 0.00875 | 0.00547 | -0.14151 | 0.16919 |
| *Cherleria biflora* | Plantae | Tracheophyta | 28 | 104 | -0.00436 | 0.00716 | -0.12027 | 0.15947 |
| *Chiloscyphus pallescens* | Plantae | Marchantiophyta | 3 | 72 | 0.01442 | 0.00072 | 0.31824 | 0.24256 |
| *Cicerbita alpina* | Plantae | Tracheophyta | 32 | 51 | -0.01613 | 0.00624 | -0.1219 | 0.1139 |
| *Circaea alpina* | Plantae | Tracheophyta | 24 | 64 | 0.00751 | 0.00735 | -0.0599 | 0.14631 |
| *Cirriphyllum piliferum* | Plantae | Bryophyta | 5 | 65 | 0.00648 | 0.005 | 0.09048 | 0.24106 |
| *Cirsium heterophyllum* | Plantae | Tracheophyta | 22 | 108 | -0.00822 | 0.00638 | -0.08663 | 0.12239 |
| *Cladonia gracilis* | Fungi | Ascomycota | 6 | 66 | 0.02629 | 0.00507 | -0.47846 | 0.50629 |
| *Clangula hyemalis* | Animalia | Chordata | 2 | 93 | -0.01905 | NA | -2.17462 | NA |
| *Clinopodium acinos* | Plantae | Tracheophyta | 78 | 120 | -0.00189 | 0.00132 | -0.04485 | 0.03725 |
| *Clinopodium vulgare* | Plantae | Tracheophyta | 25 | 59 | -0.00166 | 0.00499 | -0.22764 | 0.09079 |
| *Cloeon simile* | Animalia | Arthropoda | 8 | 57 | 0.0015 | 0.00868 | 0.05438 | 0.13924 |
| *Cochlearia officinalis* | Plantae | Tracheophyta | 11 | 53 | -0.00824 | 0.01468 | 0.231 | 0.25738 |
| *Coenagrion hastulatum* | Animalia | Arthropoda | 22 | 51 | -0.01454 | 0.00857 | -0.26647 | 0.12608 |
| *Colobanthus subulatus* | Plantae | Tracheophyta | 20 | 104 | 0.00183 | 0.00183 | -0.04734 | 0.03971 |
| *Comarum palustre* | Plantae | Tracheophyta | 29 | 104 | -0.00899 | 0.00597 | -0.10687 | 0.11557 |
| *Comastoma tenellum* | Plantae | Tracheophyta | 3 | 49 | -0.0066 | 0.00131 | -0.16943 | 0.06186 |
| *Convallaria majalis* | Plantae | Tracheophyta | 37 | 71 | -0.00567 | 0.00523 | -0.1177 | 0.09965 |
| *Corallorhiza trifida* | Plantae | Tracheophyta | 30 | 64 | 0.00449 | 0.0056 | 0.03078 | 0.11893 |
| *Cornus suecica* | Plantae | Tracheophyta | 76 | 117 | 0.00414 | 0.00282 | 0.00952 | 0.07953 |
| *Corvus cornix* | Animalia | Chordata | 15 | 110 | -0.0107 | 0.00415 | -0.1589 | 0.11868 |
| *Corydalis intermedia* | Plantae | Tracheophyta | 2 | 66 | -0.00074 | NA | 0.24459 | NA |
| *Corylus avellana* | Plantae | Tracheophyta | 36 | 58 | -0.0046 | 0.00559 | -0.07581 | 0.11685 |
| *Cotoneaster scandinavicus* | Plantae | Tracheophyta | 12 | 63 | -0.01121 | 0.00558 | -0.20486 | 0.12733 |
| *Cratoneuron filicinum* | Plantae | Bryophyta | 9 | 100 | -0.00366 | 0.0076 | -0.18103 | 0.16856 |
| *Ctenidium molluscum* | Plantae | Bryophyta | 11 | 61 | 0.00625 | 0.00568 | 0.21659 | 0.09472 |
| *Cymatia bonsdorffii* | Animalia | Arthropoda | 14 | 50 | 0.00095 | 0.00972 | 0.15011 | 0.17802 |
| *Cyrnus flavidus* | Animalia | Arthropoda | 11 | 56 | -0.0007 | 0.00457 | -0.0805 | 0.06249 |
| *Cystopteris fragilis* | Plantae | Tracheophyta | 44 | 57 | 0.01068 | 0.00568 | -0.19226 | 0.11835 |
| *Cystopteris montana* | Plantae | Tracheophyta | 7 | 54 | 0.00259 | 0.00761 | 0.63977 | 0.25045 |
| *Dactylis glomerata* | Plantae | Tracheophyta | 28 | 70 | 0.00196 | 0.00638 | -0.32845 | 0.09327 |
| *Dactylorhiza incarnata* | Plantae | Tracheophyta | 61 | 82 | 0.00066 | 0.00335 | -0.10657 | 0.07427 |
| *Dactylorhiza maculata* | Plantae | Tracheophyta | 59 | 94 | 0.01232 | 0.00431 | 0.1014 | 0.10808 |
| *Danthonia decumbens* | Plantae | Tracheophyta | 15 | 59 | 0.01435 | 0.00834 | 0.1499 | 0.17127 |
| *Daphne mezereum* | Plantae | Tracheophyta | 26 | 66 | -0.0141 | 0.00835 | -0.37152 | 0.19181 |
| *Dendronotus frondosus* | Animalia | Mollusca | 4 | 64 | 0.00649 | 0.01016 | -0.69741 | 0.94696 |
| *Deschampsia atropurpurea* | Plantae | Tracheophyta | 10 | 54 | -0.01049 | 0.03071 | -0.98263 | 0.57369 |
| *Deschampsia cespitosa* | Plantae | Tracheophyta | 39 | 66 | -0.00944 | 0.00822 | -0.25955 | 0.14599 |
| *Diapensia lapponica* | Plantae | Tracheophyta | 25 | 50 | -0.02618 | 0.00839 | -0.38983 | 0.17091 |
| *Dichodontium pellucidum* | Plantae | Bryophyta | 5 | 98 | 0.00796 | 0.00754 | 0.24447 | 0.2845 |
| *Dicranella cerviculata* | Plantae | Bryophyta | 2 | 60 | -0.01198 | NA | -0.66109 | NA |
| *Dicranum elongatum* | Plantae | Bryophyta | 6 | 74 | 0.00658 | 0.00749 | -0.12667 | 0.33501 |
| *Dicranum fragilifolium* | Plantae | Bryophyta | 2 | 103 | 0.01467 | NA | 0.39919 | NA |
| *Dicranum fuscescens* | Plantae | Bryophyta | 12 | 101 | 0.00873 | 0.01182 | 0.25242 | 0.30913 |
| *Dicranum majus* | Plantae | Bryophyta | 13 | 65 | -0.00736 | 0.01298 | -0.03114 | 0.27438 |
| *Dicranum scoparium* | Plantae | Bryophyta | 20 | 91 | 0.00241 | 0.00823 | 0.45284 | 0.23793 |
| *Dicranum undulatum* | Plantae | Bryophyta | 3 | 73 | 0.02219 | 0.00435 | 2.38415 | 0.21014 |
| *Digitalis purpurea* | Plantae | Tracheophyta | 5 | 52 | 0.00356 | 0.0058 | 0.13753 | 0.16341 |
| *Diphasiastrum complanatum* | Plantae | Tracheophyta | 4 | 66 | -0.01065 | 0.02193 | -0.85202 | 0.19252 |
| *Diplophyllum albicans* | Plantae | Marchantiophyta | 12 | 101 | 0.00879 | 0.00552 | 0.34595 | 0.15283 |
| *Distichium capillaceum* | Plantae | Bryophyta | 14 | 104 | 0.00137 | 0.00773 | 0.18066 | 0.16714 |
| *Diura nanseni* | Animalia | Arthropoda | 47 | 49 | -0.01508 | 0.00632 | -0.11053 | 0.08964 |
| *Doris pseudoargus* | Animalia | Mollusca | 8 | 70 | -0.00026 | 0.00539 | 0.12094 | 0.31404 |
| *Doto coronata* | Animalia | Mollusca | 2 | 82 | -0.01285 | NA | -0.47469 | NA |
| *Draba alpina* | Plantae | Tracheophyta | 12 | 61 | -0.0033 | 0.00208 | 0.00272 | 0.05382 |
| *Draba incana* | Plantae | Tracheophyta | 28 | 59 | 0.0316 | 0.0052 | 0.10676 | 0.17699 |
| *Draba verna* | Plantae | Tracheophyta | 9 | 61 | 0.00622 | 0.00264 | 0.0122 | 0.08155 |
| *Dryas octopetala* | Plantae | Tracheophyta | 32 | 68 | -0.01938 | 0.00876 | -0.26364 | 0.1876 |
| *Dryopteris expansa* | Plantae | Tracheophyta | 38 | 104 | 0.00018 | 0.00453 | -0.10419 | 0.10357 |
| *Dryopteris filix-mas* | Plantae | Tracheophyta | 39 | 65 | 0.00828 | 0.00498 | -0.11471 | 0.10401 |
| *Eana osseana* | Animalia | Arthropoda | 7 | 53 | 0.01544 | 0.01269 | 0.29448 | 0.19155 |
| *Eleocharis acicularis* | Plantae | Tracheophyta | 2 | 54 | -0.00691 | NA | -0.24975 | NA |
| *Eleocharis quinqueflora* | Plantae | Tracheophyta | 34 | 68 | 0.0122 | 0.00662 | -0.06985 | 0.14006 |
| *Eleocharis uniglumis* | Plantae | Tracheophyta | 17 | 71 | 0.01431 | 0.00722 | -0.09951 | 0.18748 |
| *Elymus caninus* | Plantae | Tracheophyta | 35 | 70 | -0.00526 | 0.00555 | -0.141 | 0.11451 |
| *Elymus macrourus* | Plantae | Tracheophyta | 31 | 78 | 0.00287 | 0.00779 | -0.18697 | 0.16613 |
| *Elymus repens* | Plantae | Tracheophyta | 26 | 76 | 0.00012 | 0.00619 | -0.1679 | 0.12727 |
| *Empetrum nigrum* | Plantae | Tracheophyta | 38 | 120 | -0.01516 | 0.00579 | -0.27466 | 0.1248 |
| *Enallagma cyathigerum* | Animalia | Arthropoda | 22 | 70 | 0.00137 | 0.00617 | 0.00435 | 0.14373 |
| *Ephemera vulgata* | Animalia | Arthropoda | 7 | 53 | 0.00454 | 0.00809 | 0.0146 | 0.13374 |
| *Ephemerum serratum* | Plantae | Bryophyta | 2 | 90 | 0.00278 | NA | 0.12914 | NA |
| *Epilobium alsinifolium* | Plantae | Tracheophyta | 18 | 58 | -0.00678 | 0.01185 | -0.19786 | 0.1805 |
| *Epilobium anagallidifolium* | Plantae | Tracheophyta | 26 | 63 | -0.00415 | 0.01076 | -0.09289 | 0.20524 |
| *Epilobium collinum* | Plantae | Tracheophyta | 24 | 62 | -0.00294 | 0.0058 | -0.15028 | 0.10962 |
| *Epilobium davuricum* | Plantae | Tracheophyta | 16 | 55 | 0.02478 | 0.01221 | -0.35119 | 0.18591 |
| *Epilobium hornemannii* | Plantae | Tracheophyta | 30 | 62 | 0.00359 | 0.00895 | -0.06723 | 0.16189 |
| *Epilobium lactiflorum* | Plantae | Tracheophyta | 18 | 58 | -0.00513 | 0.01184 | -0.56556 | 0.20776 |
| *Epilobium montanum* | Plantae | Tracheophyta | 39 | 86 | 0.00031 | 0.00454 | -0.14911 | 0.09619 |
| *Epilobium palustre* | Plantae | Tracheophyta | 30 | 65 | 0.01392 | 0.00748 | -0.18346 | 0.15741 |
| *Epipactis atrorubens* | Plantae | Tracheophyta | 19 | 68 | 0.00315 | 0.00501 | 0.05286 | 0.10854 |
| *Epipactis helleborine* | Plantae | Tracheophyta | 11 | 53 | 0.01695 | 0.01148 | 0.24041 | 0.22377 |
| *Equisetum arvense* | Plantae | Tracheophyta | 33 | 67 | -0.01321 | 0.0077 | -0.36808 | 0.12408 |
| *Equisetum palustre* | Plantae | Tracheophyta | 31 | 66 | 0.0036 | 0.00689 | -0.19435 | 0.12576 |
| *Equisetum pratense* | Plantae | Tracheophyta | 24 | 66 | -0.00514 | 0.00972 | -0.49606 | 0.15725 |
| *Equisetum variegatum* | Plantae | Tracheophyta | 36 | 63 | -0.00176 | 0.00882 | -0.21881 | 0.16986 |
| *Erica tetralix* | Plantae | Tracheophyta | 30 | 55 | 0.02517 | 0.00564 | 0.16858 | 0.14062 |
| *Erigeron borealis* | Plantae | Tracheophyta | 33 | 80 | -0.01761 | 0.00792 | -0.46423 | 0.1723 |
| *Erigeron politus* | Plantae | Tracheophyta | 42 | 116 | 0.00076 | 0.0024 | 0.05245 | 0.06506 |
| *Erigeron uniflorus* | Plantae | Tracheophyta | 28 | 63 | 0.01264 | 0.00956 | -0.21832 | 0.17743 |
| *Eriophorum angustifolium* | Plantae | Tracheophyta | 37 | 70 | -0.01495 | 0.008 | -0.38323 | 0.14522 |
| *Eriophorum latifolium* | Plantae | Tracheophyta | 49 | 66 | 0.0079 | 0.00419 | -0.02231 | 0.09543 |
| *Eriophorum scheuchzeri* | Plantae | Tracheophyta | 24 | 60 | -0.01661 | 0.01129 | -0.33871 | 0.22969 |
| *Eriophorum vaginatum* | Plantae | Tracheophyta | 30 | 104 | -0.01206 | 0.00669 | -0.22117 | 0.13676 |
| *Eulithis populata* | Animalia | Arthropoda | 8 | 50 | -0.03406 | 0.02701 | 0.71807 | 0.52807 |
| *Euphrasia frigida* | Plantae | Tracheophyta | 31 | 55 | -0.0046 | 0.01022 | -0.0046 | 0.19613 |
| *Euphrasia stricta* | Plantae | Tracheophyta | 28 | 98 | -0.00223 | 0.00469 | -0.04969 | 0.12789 |
| *Euphrasia vernalis* | Plantae | Tracheophyta | 14 | 104 | 0.00399 | 0.00643 | 0.14176 | 0.14853 |
| *Facelina auriculata* | Animalia | Mollusca | 3 | 64 | 0.00239 | 0.00183 | -0.00346 | 0.22768 |
| *Falco columbarius* | Animalia | Chordata | 9 | 55 | 0.0235 | 0.0099 | 0.11913 | 0.33618 |
| *Fallopia convolvulus* | Plantae | Tracheophyta | 4 | 57 | -0.00873 | 0.0122 | 0.33133 | 0.99011 |
| *Festuca ovina* | Plantae | Tracheophyta | 30 | 56 | -0.00883 | 0.01015 | -0.24467 | 0.19487 |
| *Festuca richardsonii* | Plantae | Tracheophyta | 17 | 71 | 0.00763 | 0.00514 | 0.20387 | 0.14416 |
| *Festuca rubra* | Plantae | Tracheophyta | 46 | 70 | -0.00773 | 0.00508 | -0.32373 | 0.11395 |
| *Festuca vivipara* | Plantae | Tracheophyta | 41 | 56 | 0.00002 | 0.00709 | 0.03917 | 0.13194 |
| *Ficedula hypoleuca* | Animalia | Chordata | 4 | 64 | 0.01801 | 0.01162 | 0.51021 | 0.33634 |
| *Fissidens exilis* | Plantae | Bryophyta | 2 | 95 | -0.00017 | NA | -0.00683 | NA |
| *Fissidens osmundoides* | Plantae | Bryophyta | 12 | 99 | 0.01076 | 0.00547 | 0.42984 | 0.16844 |
| *Fissidens viridulus* | Plantae | Bryophyta | 3 | 95 | -0.00092 | 0.00071 | -0.03458 | 0.03453 |
| *Flexitrichum flexicaule* | Plantae | Bryophyta | 16 | 102 | 0.01283 | 0.00652 | 0.48709 | 0.18334 |
| *Flexitrichum gracile* | Plantae | Bryophyta | 7 | 98 | 0.00932 | 0.00698 | 0.50554 | 0.20962 |
| *Fomitopsis betulina* | Fungi | Basidiomycota | 4 | 85 | 0.01367 | 0.00431 | 0.25478 | 0.60403 |
| *Frangula alnus* | Plantae | Tracheophyta | 5 | 51 | -0.0182 | 0.01033 | 0.2799 | 0.29338 |
| *Fringilla coelebs* | Animalia | Chordata | 9 | 94 | -0.00143 | 0.00313 | 0.10319 | 0.10697 |
| *Fringilla montifringilla* | Animalia | Chordata | 12 | 107 | -0.00981 | 0.00316 | -0.44225 | 0.13281 |
| *Frullania tamarisci* | Plantae | Marchantiophyta | 9 | 108 | 0.00502 | 0.00759 | 0.34285 | 0.22926 |
| *Fumaria officinalis* | Plantae | Tracheophyta | 8 | 63 | 0.00499 | 0.00944 | -0.24801 | 0.1594 |
| *Fuscocephaloziopsis albescens* | Plantae | Marchantiophyta | 3 | 57 | -0.00134 | 0.00119 | -0.04625 | 0.04197 |
| *Fuscocephaloziopsis lunulifolia* | Plantae | Marchantiophyta | 9 | 84 | 0.01191 | 0.00486 | 0.18451 | 0.19087 |
| *Gagea lutea* | Plantae | Tracheophyta | 15 | 53 | 0.00783 | 0.00491 | 0.01853 | 0.10749 |
| *Galba truncatula* | Animalia | Mollusca | 2 | 89 | -0.0111 | NA | -0.4929 | NA |
| *Galeopsis tetrahit* | Plantae | Tracheophyta | 12 | 53 | 0.01048 | 0.01369 | -0.22848 | 0.25338 |
| *Galium boreale* | Plantae | Tracheophyta | 32 | 59 | -0.01026 | 0.00752 | -0.36449 | 0.10296 |
| *Galium odoratum* | Plantae | Tracheophyta | 33 | 66 | -0.00255 | 0.0054 | -0.13604 | 0.10979 |
| *Galium palustre* | Plantae | Tracheophyta | 37 | 67 | 0.0045 | 0.00615 | -0.19547 | 0.1161 |
| *Galium verum* | Plantae | Tracheophyta | 19 | 53 | -0.00119 | 0.00852 | -0.11986 | 0.12753 |
| *Gavia arctica* | Animalia | Chordata | 5 | 108 | -0.00643 | 0.00316 | -0.32033 | 0.08495 |
| *Gavia stellata* | Animalia | Chordata | 4 | 107 | -0.02003 | 0.01016 | -0.7307 | 0.6545 |
| *Gentiana nivalis* | Plantae | Tracheophyta | 13 | 55 | -0.02232 | 0.01631 | -0.79671 | 0.2289 |
| *Gentiana purpurea* | Plantae | Tracheophyta | 2 | 64 | -0.00021 | NA | 0.10074 | NA |
| *Gentianella amarella* | Plantae | Tracheophyta | 11 | 65 | 0.00357 | 0.01478 | -0.10076 | 0.2185 |
| *Gentianella campestris* | Plantae | Tracheophyta | 29 | 71 | 0.01653 | 0.00622 | -0.06007 | 0.15906 |
| *Geranium sylvaticum* | Plantae | Tracheophyta | 35 | 60 | -0.02163 | 0.00849 | -0.31083 | 0.14559 |
| *Geum rivale* | Plantae | Tracheophyta | 32 | 54 | -0.02122 | 0.00811 | -0.31535 | 0.13077 |
| *Geum urbanum* | Plantae | Tracheophyta | 28 | 55 | -0.00542 | 0.0051 | -0.04312 | 0.09342 |
| *Gibbula tumida* | Animalia | Mollusca | 6 | 62 | -0.001 | 0.00552 | -0.02287 | 0.27414 |
| *Glaucidium passerinum* | Animalia | Chordata | 9 | 61 | 0.00042 | 0.00483 | -0.09045 | 0.07808 |
| *Glossiphonia complanata* | Animalia | Annelida | 13 | 53 | -0.0119 | 0.00793 | -0.27187 | 0.15425 |
| *Glyceria fluitans* | Plantae | Tracheophyta | 19 | 52 | 0.00287 | 0.00812 | -0.03906 | 0.12638 |
| *Grimmia funalis* | Plantae | Bryophyta | 5 | 71 | -0.00475 | 0.00049 | -0.12668 | 0.05036 |
| *Grimmia ramondii* | Plantae | Bryophyta | 4 | 71 | 0.00855 | 0.00978 | -0.20398 | 0.47601 |
| *Grimmia torquata* | Plantae | Bryophyta | 6 | 101 | 0.01104 | 0.0091 | 0.52979 | 0.25803 |
| *Gymnadenia conopsea* | Plantae | Tracheophyta | 31 | 68 | 0.01139 | 0.00749 | 0.16962 | 0.13527 |
| *Gymnadenia nigra* | Plantae | Tracheophyta | 5 | 69 | -0.00268 | 0.00008 | -0.05803 | 0.04579 |
| *Gymnocolea inflata* | Plantae | Marchantiophyta | 4 | 92 | 0.01587 | 0.0077 | 0.47687 | 0.20327 |
| *Gymnomitrion concinnatum* | Plantae | Marchantiophyta | 5 | 109 | 0.01437 | 0.00875 | 0.4647 | 0.158 |
| *Gymnostomum aeruginosum* | Plantae | Bryophyta | 10 | 111 | 0.0113 | 0.00258 | 0.32419 | 0.07513 |
| *Gyraulus acronicus* | Animalia | Mollusca | 33 | 92 | -0.01114 | 0.00308 | -0.13963 | 0.09021 |
| *Haematopus ostralegus* | Animalia | Chordata | 4 | 82 | -0.02244 | 0.00769 | -0.44989 | 0.22346 |
| *Halesus radiatus* | Animalia | Arthropoda | 9 | 50 | -0.00093 | 0.01043 | 0.01997 | 0.12064 |
| *Haliplus confinis* | Animalia | Arthropoda | 7 | 108 | -0.00188 | 0.0021 | 0.00925 | 0.06853 |
| *Hammarbya paludosa* | Plantae | Tracheophyta | 7 | 56 | 0.02567 | 0.01637 | 1.24255 | 0.34648 |
| *Harpanthus flotovianus* | Plantae | Marchantiophyta | 4 | 91 | 0.01496 | 0.00507 | 0.79606 | 0.32124 |
| *Helobdella stagnalis* | Animalia | Annelida | 23 | 52 | -0.00626 | 0.00607 | -0.03152 | 0.12059 |
| *Hemiclepsis tessulata* | Animalia | Annelida | 11 | 52 | -0.00234 | 0.01588 | -0.07354 | 0.22051 |
| *Hepatica nobilis* | Plantae | Tracheophyta | 23 | 74 | -0.00026 | 0.00309 | -0.07679 | 0.06794 |
| *Hepialus fusconebulosa* | Animalia | Arthropoda | 7 | 50 | -0.00153 | 0.01381 | -0.11215 | 0.17754 |
| *Hesperis matronalis* | Plantae | Tracheophyta | 8 | 72 | 0.00631 | 0.0054 | 0.01254 | 0.15309 |
| *Hieracium caesium* | Plantae | Tracheophyta | 11 | 62 | 0.00703 | 0.01072 | -0.02098 | 0.27115 |
| *Hieracium lachenalii* | Plantae | Tracheophyta | 19 | 84 | -0.0021 | 0.00536 | -0.04003 | 0.13723 |
| *Hippophae rhamnoides* | Plantae | Tracheophyta | 10 | 79 | -0.00084 | 0.00244 | -0.10241 | 0.05815 |
| *Holcus lanatus* | Plantae | Tracheophyta | 13 | 55 | 0.01743 | 0.00645 | 0.21866 | 0.17786 |
| *Holocentropus picicornis* | Animalia | Arthropoda | 9 | 52 | -0.00336 | 0.00581 | 0.02361 | 0.11283 |
| *Humulus lupulus* | Plantae | Tracheophyta | 9 | 52 | -0.00013 | 0.01103 | 0.10546 | 0.26634 |
| *Huperzia selago* | Plantae | Tracheophyta | 31 | 49 | -0.02142 | 0.00883 | -0.23358 | 0.14099 |
| *Hydroporus palustris* | Animalia | Arthropoda | 23 | 49 | 0.00314 | 0.00857 | 0.23337 | 0.16542 |
| *Hygrocybe russocoriacea* | Fungi | Basidiomycota | 14 | 50 | -0.00811 | 0.01126 | -0.02471 | 0.15707 |
| *Hylesinus varius* | Animalia | Arthropoda | 2 | 89 | -0.01284 | NA | -0.34537 | NA |
| *Hylocomium splendens* | Plantae | Bryophyta | 15 | 64 | 0.00032 | 0.01272 | 0.26163 | 0.2607 |
| *Hymenoloma crispulum* | Plantae | Bryophyta | 3 | 62 | -0.01876 | 0.00277 | -0.49322 | 0.27604 |
| *Hypericum hirsutum* | Plantae | Tracheophyta | 26 | 70 | 0.00622 | 0.00562 | -0.10112 | 0.12037 |
| *Hypnum callichroum* | Plantae | Bryophyta | 22 | 111 | -0.00078 | 0.00437 | -0.06955 | 0.11077 |
| *Hypnum cupressiforme* | Plantae | Bryophyta | 16 | 108 | 0.00366 | 0.00529 | 0.15486 | 0.13954 |
| *Hypnum hamulosum* | Plantae | Bryophyta | 27 | 117 | 0.00195 | 0.00459 | -0.1235 | 0.11305 |
| *Hypnum revolutum* | Plantae | Bryophyta | 16 | 120 | 0.00409 | 0.00329 | 0.14283 | 0.08906 |
| *Hypogymnia physodes* | Fungi | Ascomycota | 6 | 52 | 0.01503 | 0.00766 | -0.02977 | 0.27476 |
| *Hypopitys monotropa* | Plantae | Tracheophyta | 24 | 115 | 0.00366 | 0.00299 | 0.0683 | 0.08673 |
| *Ilybius fuliginosus* | Animalia | Arthropoda | 21 | 49 | 0.01341 | 0.0088 | 0.22804 | 0.17601 |
| *Impatiens noli-tangere* | Plantae | Tracheophyta | 16 | 53 | -0.00451 | 0.0066 | -0.01658 | 0.13619 |
| *Iothia fulva* | Animalia | Mollusca | 6 | 50 | 0.00535 | 0.00187 | 0.16947 | 0.05443 |
| *Isopterygiopsis pulchella* | Plantae | Bryophyta | 6 | 76 | 0.00666 | 0.00628 | 0.15829 | 0.17769 |
| *Isothecium alopecuroides* | Plantae | Bryophyta | 6 | 91 | 0.00378 | 0.00414 | 0.04066 | 0.14466 |
| *Judolia montivagans* | Animalia | Arthropoda | 3 | 66 | 0.02237 | 0.01525 | 0.42215 | 1.1692 |
| *Juncus alpinoarticulatus* | Plantae | Tracheophyta | 43 | 58 | 0.00437 | 0.0063 | -0.18946 | 0.12257 |
| *Juncus arcticus* | Plantae | Tracheophyta | 5 | 51 | 0.02224 | 0.01627 | 0.15877 | 0.33022 |
| *Juncus articulatus* | Plantae | Tracheophyta | 37 | 58 | 0.00599 | 0.00571 | -0.14999 | 0.113 |
| *Juncus bufonius* | Plantae | Tracheophyta | 18 | 53 | -0.00021 | 0.00976 | -0.30843 | 0.13822 |
| *Juncus bulbosus* | Plantae | Tracheophyta | 35 | 59 | 0.00664 | 0.0064 | 0.01892 | 0.11723 |
| *Juncus castaneus* | Plantae | Tracheophyta | 32 | 60 | -0.01231 | 0.00678 | -0.21838 | 0.13219 |
| *Juncus conglomeratus* | Plantae | Tracheophyta | 19 | 49 | 0.01756 | 0.00906 | 0.08867 | 0.14489 |
| *Juncus effusus* | Plantae | Tracheophyta | 32 | 53 | 0.01007 | 0.00622 | -0.04511 | 0.10986 |
| *Juncus gerardii* | Plantae | Tracheophyta | 16 | 75 | 0.00557 | 0.00851 | -0.08067 | 0.16977 |
| *Juncus squarrosus* | Plantae | Tracheophyta | 13 | 49 | -0.00689 | 0.00826 | -0.13451 | 0.13371 |
| *Juncus stygius* | Plantae | Tracheophyta | 12 | 55 | -0.0079 | 0.01083 | -0.33094 | 0.15792 |
| *Juncus triglumis* | Plantae | Tracheophyta | 32 | 63 | -0.00431 | 0.00914 | -0.11731 | 0.18261 |
| *Jungermannia eucordifolia* | Plantae | Marchantiophyta | 2 | 65 | 0.01198 | NA | 0.47421 | NA |
| *Kalmia procumbens* | Plantae | Tracheophyta | 31 | 49 | -0.03126 | 0.00986 | -0.27773 | 0.17928 |
| *Knautia arvensis* | Plantae | Tracheophyta | 22 | 56 | 0.00574 | 0.00761 | -0.10592 | 0.1221 |
| *Koenigia islandica* | Plantae | Tracheophyta | 10 | 63 | 0.00798 | 0.01655 | 0.22157 | 0.29625 |
| *Lagopus lagopus* | Animalia | Chordata | 8 | 108 | -0.01425 | 0.00475 | -0.16473 | 0.12082 |
| *Lapsana communis* | Plantae | Tracheophyta | 14 | 53 | -0.00974 | 0.00797 | -0.0739 | 0.1561 |
| *Larus argentatus* | Animalia | Chordata | 6 | 82 | -0.02105 | 0.00608 | -0.40275 | 0.1517 |
| *Larus canus* | Animalia | Chordata | 12 | 104 | -0.01568 | 0.00735 | -0.43314 | 0.18103 |
| *Larus fuscus* | Animalia | Chordata | 5 | 83 | -0.01157 | 0.01207 | -0.46527 | 0.2467 |
| *Larus marinus* | Animalia | Chordata | 7 | 83 | -0.02353 | 0.00312 | -0.53207 | 0.1941 |
| *Lathyrus pratensis* | Plantae | Tracheophyta | 25 | 59 | -0.0035 | 0.01033 | -0.2373 | 0.14989 |
| *Lathyrus vernus* | Plantae | Tracheophyta | 30 | 73 | 0.00586 | 0.00561 | -0.21272 | 0.10295 |
| *Lejeunea cavifolia* | Plantae | Marchantiophyta | 5 | 96 | 0.00248 | 0.00344 | 0.06147 | 0.10514 |
| *Lemmus lemmus* | Animalia | Chordata | 8 | 103 | -0.00905 | 0.00548 | -0.28177 | 0.17062 |
| *Lentinus substrictus* | Fungi | Basidiomycota | 10 | 68 | 0.02635 | 0.00798 | 0.50287 | 0.22667 |
| *Lepeta caeca* | Animalia | Mollusca | 5 | 89 | -0.00076 | 0.00547 | -0.05575 | 0.11831 |
| *Lepidostoma hirtum* | Animalia | Arthropoda | 27 | 58 | -0.00598 | 0.00631 | 0.00849 | 0.07676 |
| *Lepidozia reptans* | Plantae | Marchantiophyta | 7 | 87 | 0.01058 | 0.00544 | 0.15319 | 0.24104 |
| *Leptophlebia vespertina* | Animalia | Arthropoda | 25 | 53 | -0.00756 | 0.00745 | -0.1055 | 0.11204 |
| *Lescuraea incurvata* | Plantae | Bryophyta | 3 | 82 | 0.02279 | 0.0007 | 0.61434 | 0.50047 |
| *Lescuraea saxicola* | Plantae | Bryophyta | 2 | 63 | -0.00137 | NA | -0.13838 | NA |
| *Limnephilus borealis* | Animalia | Arthropoda | 7 | 68 | -0.00136 | 0.00739 | 0.22677 | 0.19376 |
| *Limnephilus flavicornis* | Animalia | Arthropoda | 3 | 52 | 0.00392 | 0.01368 | -0.00738 | 0.22399 |
| *Limnephilus nigriceps* | Animalia | Arthropoda | 8 | 69 | 0.00035 | 0.00565 | 0.10662 | 0.10501 |
| *Limnephilus rhombicus* | Animalia | Arthropoda | 6 | 70 | -0.01138 | 0.01282 | 0.25479 | 0.2712 |
| *Limnephilus stigma* | Animalia | Arthropoda | 5 | 55 | -0.00045 | 0.0119 | 0.13886 | 0.28453 |
| *Linum catharticum* | Plantae | Tracheophyta | 32 | 80 | 0.01032 | 0.00421 | 0.06368 | 0.10521 |
| *Loeskypnum badium* | Plantae | Bryophyta | 2 | 69 | 0.01651 | NA | 2.23549 | NA |
| *Lolium arundinaceum* | Plantae | Tracheophyta | 14 | 118 | 0.00034 | 0.00448 | 0.01811 | 0.10342 |
| *Lolium giganteum* | Plantae | Tracheophyta | 15 | 54 | -0.00277 | 0.00315 | -0.00063 | 0.05475 |
| *Lolium pratense* | Plantae | Tracheophyta | 71 | 117 | 0.0027 | 0.00246 | -0.04679 | 0.06865 |
| *Lophocolea bidentata* | Plantae | Marchantiophyta | 3 | 101 | 0.00139 | 0.00569 | 0.27634 | 0.15725 |
| *Lophozia ventricosa* | Plantae | Marchantiophyta | 8 | 101 | 0.01057 | 0.00705 | 0.68027 | 0.24683 |
| *Lotus corniculatus* | Plantae | Tracheophyta | 40 | 55 | 0.00323 | 0.0073 | -0.19582 | 0.12279 |
| *Luzula arcuata* | Plantae | Tracheophyta | 20 | 59 | -0.01508 | 0.00754 | -0.41091 | 0.16898 |
| *Luzula multiflora* | Plantae | Tracheophyta | 59 | 67 | 0.00038 | 0.0048 | -0.14305 | 0.10664 |
| *Luzula pilosa* | Plantae | Tracheophyta | 32 | 55 | -0.01793 | 0.00868 | -0.35629 | 0.12719 |
| *Luzula spicata* | Plantae | Tracheophyta | 36 | 70 | -0.01938 | 0.00818 | -0.49914 | 0.16212 |
| *Luzula sudetica* | Plantae | Tracheophyta | 43 | 66 | 0.00712 | 0.00718 | -0.33234 | 0.13301 |
| *Luzula sylvatica* | Plantae | Tracheophyta | 21 | 55 | 0.00653 | 0.00786 | 0.15156 | 0.16217 |
| *Lycopodium lagopus* | Plantae | Tracheophyta | 25 | 99 | -0.00419 | 0.00356 | -0.10603 | 0.07977 |
| *Lyrurus tetrix* | Animalia | Chordata | 5 | 81 | -0.00468 | 0.00321 | -0.12058 | 0.20403 |
| *Malus pumila* | Plantae | Tracheophyta | 10 | 91 | -0.00261 | 0.00219 | -0.10741 | 0.10799 |
| *Marchantia quadrata* | Plantae | Marchantiophyta | 6 | 108 | -0.00331 | 0.0063 | 0.0149 | 0.19086 |
| *Margarites groenlandicus* | Animalia | Mollusca | 8 | 62 | 0.00074 | 0.00386 | 0.04996 | 0.08233 |
| *Margarites helicinus* | Animalia | Mollusca | 6 | 101 | -0.00266 | 0.00337 | -0.10601 | 0.09937 |
| *Marsupella emarginata* | Plantae | Marchantiophyta | 7 | 97 | 0.01007 | 0.0048 | 0.35009 | 0.1265 |
| *Matricaria discoidea* | Plantae | Tracheophyta | 68 | 112 | 0.00057 | 0.00266 | -0.10597 | 0.07463 |
| *Matteuccia struthiopteris* | Plantae | Tracheophyta | 21 | 49 | -0.02168 | 0.00969 | -0.36449 | 0.13186 |
| *Megarthrus depressus* | Animalia | Arthropoda | 2 | 61 | -0.0047 | NA | 1.1497 | NA |
| *Melampyrum pratense* | Plantae | Tracheophyta | 30 | 55 | -0.01653 | 0.00904 | -0.20808 | 0.14064 |
| *Melica nutans* | Plantae | Tracheophyta | 32 | 55 | -0.00443 | 0.0078 | -0.19859 | 0.12444 |
| *Mentha arvensis* | Plantae | Tracheophyta | 16 | 58 | 0.00979 | 0.00786 | 0.03969 | 0.14028 |
| *Mergus serrator* | Animalia | Chordata | 3 | 50 | -0.02039 | 0.03575 | -0.29594 | 0.68524 |
| *Mesoptychia bantriensis* | Plantae | Marchantiophyta | 4 | 112 | 0.01417 | 0.00359 | 0.50748 | 0.08542 |
| *Mesoptychia collaris* | Plantae | Marchantiophyta | 2 | 65 | 0.00486 | NA | 3.34123 | NA |
| *Mesotype didymata* | Animalia | Arthropoda | 2 | 57 | -0.00015 | NA | -0.00478 | NA |
| *Micranthes stellaris* | Plantae | Tracheophyta | 35 | 67 | -0.00837 | 0.00845 | -0.31441 | 0.17566 |
| *Micranthes tenuis* | Plantae | Tracheophyta | 10 | 57 | 0.02271 | 0.01693 | 0.13777 | 0.2894 |
| *Microtus agrestis* | Animalia | Chordata | 4 | 93 | 0.01037 | 0.00935 | 0.22686 | 0.26341 |
| *Milium effusum* | Plantae | Tracheophyta | 25 | 54 | -0.00144 | 0.00767 | -0.23541 | 0.13359 |
| *Mnium spinosum* | Plantae | Bryophyta | 3 | 73 | 0.00005 | 0.00742 | -0.20745 | 0.32062 |
| *Moehringia trinervia* | Plantae | Tracheophyta | 23 | 54 | 0.01253 | 0.00554 | 0.09273 | 0.12652 |
| *Molanna angustata* | Animalia | Arthropoda | 3 | 50 | 0.00276 | 0.00112 | 0.04416 | 0.00224 |
| *Molannodes tinctus* | Animalia | Arthropoda | 8 | 68 | -0.00232 | 0.00769 | 0.03613 | 0.21409 |
| *Molinia caerulea* | Plantae | Tracheophyta | 33 | 104 | -0.00824 | 0.00579 | -0.26805 | 0.11664 |
| *Moneses uniflora* | Plantae | Tracheophyta | 17 | 93 | -0.00111 | 0.00709 | -0.16641 | 0.14604 |
| *Mus musculus* | Animalia | Chordata | 5 | 50 | -0.0047 | 0.00285 | -0.17057 | 0.21249 |
| *Mycetoporus lepidus* | Animalia | Arthropoda | 2 | 61 | -0.00102 | NA | 0.24899 | NA |
| *Mycoblastus sanguinarius* | Fungi | Ascomycota | 2 | 64 | 0.00883 | NA | 0.91423 | NA |
| *Mylia taylorii* | Plantae | Marchantiophyta | 19 | 105 | 0.0073 | 0.00336 | 0.1741 | 0.09809 |
| *Myodes glareolus* | Animalia | Chordata | 7 | 59 | 0.01553 | 0.00687 | 0.2964 | 0.27133 |
| *Myosotis arvensis* | Plantae | Tracheophyta | 36 | 70 | 0.00147 | 0.00439 | -0.16329 | 0.10165 |
| *Myosotis decumbens* | Plantae | Tracheophyta | 14 | 49 | -0.00618 | 0.01241 | -0.49405 | 0.22381 |
| *Myosotis laxa* | Plantae | Tracheophyta | 12 | 52 | 0.01442 | 0.00722 | -0.08167 | 0.15762 |
| *Myrica gale* | Plantae | Tracheophyta | 20 | 50 | 0.0167 | 0.01101 | -0.13646 | 0.19052 |
| *Myricaria germanica* | Plantae | Tracheophyta | 14 | 79 | 0.00268 | 0.00496 | -0.05773 | 0.11563 |
| *Myriophyllum alterniflorum* | Plantae | Tracheophyta | 21 | 58 | -0.00751 | 0.0076 | -0.02529 | 0.12826 |
| *Mystacides azureus* | Animalia | Arthropoda | 15 | 68 | 0.00201 | 0.00507 | 0.01626 | 0.10551 |
| *Myurella julacea* | Plantae | Bryophyta | 4 | 76 | 0.00367 | 0.01287 | -0.05035 | 0.38593 |
| *Myurella tenerrima* | Plantae | Bryophyta | 6 | 110 | 0.00536 | 0.00593 | 0.0789 | 0.2292 |
| *Nardia geoscyphus* | Plantae | Marchantiophyta | 4 | 78 | 0.00081 | 0.00301 | -0.16523 | 0.03386 |
| *Nardia scalaris* | Plantae | Marchantiophyta | 2 | 73 | 0.01375 | NA | 3.04515 | NA |
| *Nardus stricta* | Plantae | Tracheophyta | 34 | 105 | -0.01807 | 0.00672 | -0.2521 | 0.13834 |
| *Narthecium ossifragum* | Plantae | Tracheophyta | 45 | 71 | 0.0042 | 0.00573 | -0.06572 | 0.12121 |
| *Nebrioporus depressus* | Animalia | Arthropoda | 12 | 49 | 0.0147 | 0.00737 | 0.25519 | 0.18777 |
| *Nemoura cinerea* | Animalia | Arthropoda | 32 | 69 | -0.0147 | 0.00572 | -0.11817 | 0.08637 |
| *Neoorthocaulis floerkei* | Plantae | Marchantiophyta | 4 | 62 | 0.01868 | 0.01643 | 0.11258 | 0.52365 |
| *Neottia cordata* | Plantae | Tracheophyta | 27 | 86 | 0.00833 | 0.00682 | 0.23518 | 0.13912 |
| *Neottia ovata* | Plantae | Tracheophyta | 35 | 68 | 0.00918 | 0.0055 | -0.058 | 0.10584 |
| *Neptunea despecta* | Animalia | Mollusca | 7 | 68 | 0.00172 | 0.00175 | 0.044 | 0.05395 |
| *Nigrobaetis niger* | Animalia | Arthropoda | 34 | 57 | 0.00603 | 0.00534 | 0.15358 | 0.06251 |
| *Notiophilus fasciatus* | Animalia | Arthropoda | 5 | 68 | 0.01852 | 0.01153 | 0.09962 | 1.11356 |
| *Nucella lapillus* | Animalia | Mollusca | 16 | 102 | -0.00596 | 0.00458 | -0.02537 | 0.11336 |
| *Numenius arquata* | Animalia | Chordata | 4 | 89 | 0.00201 | 0.00238 | 0.08014 | 0.11574 |
| *Ochrolechia androgyna* | Fungi | Ascomycota | 13 | 88 | 0.00599 | 0.00793 | 0.07601 | 0.17537 |
| *Ochrolechia subviridis* | Fungi | Ascomycota | 5 | 80 | -0.00566 | 0.00895 | -0.20346 | 0.23064 |
| *Odontoschisma fluitans* | Plantae | Marchantiophyta | 2 | 79 | 0.00302 | NA | 0.17099 | NA |
| *Omalotheca norvegica* | Plantae | Tracheophyta | 36 | 60 | -0.00547 | 0.00817 | -0.36767 | 0.16074 |
| *Omalotheca supina* | Plantae | Tracheophyta | 26 | 60 | -0.02604 | 0.00918 | -0.37164 | 0.21427 |
| *Omalotheca sylvatica* | Plantae | Tracheophyta | 24 | 57 | -0.00848 | 0.00659 | -0.27452 | 0.11254 |
| *Onchidoris muricata* | Animalia | Mollusca | 8 | 92 | 0.0011 | 0.00258 | 0.01688 | 0.09148 |
| *Oncophorus virens* | Plantae | Bryophyta | 6 | 77 | 0.00449 | 0.00904 | 0.11048 | 0.45009 |
| *Oreopteris limbosperma* | Plantae | Tracheophyta | 33 | 65 | -0.00464 | 0.00507 | -0.21194 | 0.08379 |
| *Origanum vulgare* | Plantae | Tracheophyta | 4 | 58 | 0.00197 | 0.01197 | 0.28195 | 0.1692 |
| *Orthilia secunda* | Plantae | Tracheophyta | 31 | 56 | 0.00287 | 0.00685 | -0.13113 | 0.11436 |
| *Orthosia gothica* | Animalia | Arthropoda | 11 | 49 | 0.00042 | 0.00265 | 0.03542 | 0.03583 |
| *Orthotrichum affine* | Plantae | Bryophyta | 4 | 75 | 0.00415 | 0.00576 | -0.65365 | 0.79383 |
| *Orthotrichum gymnostomum* | Plantae | Bryophyta | 19 | 107 | 0.0012 | 0.00463 | 0.01166 | 0.09637 |
| *Orthotrichum obtusifolium* | Plantae | Bryophyta | 21 | 113 | 0.00406 | 0.00288 | 0.1229 | 0.07425 |
| *Orthotrichum rupestre* | Plantae | Bryophyta | 22 | 112 | 0.00686 | 0.00346 | 0.26762 | 0.10999 |
| *Orthotrichum speciosum* | Plantae | Bryophyta | 39 | 115 | 0.00669 | 0.00209 | 0.12356 | 0.06582 |
| *Orthotrichum stramineum* | Plantae | Bryophyta | 5 | 106 | 0.00312 | 0.0035 | 0.16272 | 0.09216 |
| *Oxyrrhynchium hians* | Plantae | Bryophyta | 2 | 97 | 0.00288 | NA | 0.10857 | NA |
| *Oxytropis lapponica* | Plantae | Tracheophyta | 9 | 52 | -0.00384 | 0.01759 | -0.41175 | 0.29967 |
| *Papaver radicatum* | Plantae | Tracheophyta | 5 | 64 | 0.0013 | 0.00251 | 0.06487 | 0.0486 |
| *Paris quadrifolia* | Plantae | Tracheophyta | 33 | 50 | -0.00013 | 0.00609 | -0.16149 | 0.09408 |
| *Parnassia palustris* | Plantae | Tracheophyta | 31 | 60 | -0.01243 | 0.00979 | -0.13085 | 0.16188 |
| *Parus major* | Animalia | Chordata | 17 | 67 | 0.00066 | 0.00424 | 0.09052 | 0.09248 |
| *Parus montanus* | Animalia | Chordata | 22 | 58 | -0.00924 | 0.00482 | 0.15772 | 0.10495 |
| *Passer domesticus* | Animalia | Chordata | 5 | 61 | -0.01218 | 0.00227 | -0.08925 | 0.13484 |
| *Pectenia plumbea* | Fungi | Ascomycota | 6 | 72 | -0.01097 | 0.0076 | -0.11941 | 0.22496 |
| *Pedicularis oederi* | Plantae | Tracheophyta | 35 | 56 | -0.00169 | 0.00267 | -0.07553 | 0.05822 |
| *Pedicularis palustris* | Plantae | Tracheophyta | 33 | 52 | 0.00229 | 0.00786 | -0.09928 | 0.12948 |
| *Pedicularis sceptrum-carolinum* | Plantae | Tracheophyta | 17 | 50 | -0.01362 | 0.01804 | -0.02592 | 0.25608 |
| *Pedicularis sylvatica* | Plantae | Tracheophyta | 13 | 52 | -0.00308 | 0.00405 | -0.05251 | 0.06798 |
| *Peltigera canina* | Fungi | Ascomycota | 2 | 53 | 0.0154 | NA | 3.13784 | NA |
| *Peltigera collina* | Fungi | Ascomycota | 7 | 50 | 0.00255 | 0.01056 | -0.54343 | 0.28869 |
| *Peregriana peregra* | Animalia | Mollusca | 20 | 78 | -0.00375 | 0.00483 | -0.03109 | 0.13992 |
| *Persicaria lapathifolia* | Plantae | Tracheophyta | 9 | 68 | 0.01114 | 0.00942 | -0.10331 | 0.28044 |
| *Petasites frigidus* | Plantae | Tracheophyta | 17 | 56 | -0.02658 | 0.02089 | -0.19692 | 0.36686 |
| *Phalacrocorax aristotelis* | Animalia | Chordata | 6 | 81 | -0.00179 | 0.01004 | 0.01414 | 0.34012 |
| *Phalacrocorax carbo* | Animalia | Chordata | 8 | 100 | -0.01331 | 0.00627 | -0.53383 | 0.23143 |
| *Phalaris arundinacea* | Plantae | Tracheophyta | 31 | 52 | -0.00907 | 0.00732 | -0.14797 | 0.10604 |
| *Philonotis calcarea* | Plantae | Bryophyta | 4 | 104 | 0.00537 | 0.00292 | 0.15544 | 0.10661 |
| *Philonotis fontana* | Plantae | Bryophyta | 7 | 101 | 0.01138 | 0.01344 | 0.55196 | 0.36729 |
| *Philopotamus montanus* | Animalia | Arthropoda | 12 | 54 | 0.01303 | 0.01204 | 0.27707 | 0.15787 |
| *Phippsia algida* | Plantae | Tracheophyta | 8 | 70 | -0.00127 | 0.01176 | -0.12418 | 0.25138 |
| *Phippsia concinna* | Plantae | Tracheophyta | 3 | 60 | 0.00491 | 0.00182 | 0.06664 | 0.08247 |
| *Phleum alpinum* | Plantae | Tracheophyta | 31 | 54 | -0.02607 | 0.00987 | -0.33278 | 0.18859 |
| *Phoenicurus phoenicurus* | Animalia | Chordata | 2 | 64 | 0.02084 | NA | 0.5403 | NA |
| *Phragmites australis* | Plantae | Tracheophyta | 13 | 49 | 0.01204 | 0.01145 | 0.17926 | 0.13129 |
| *Phyllodoce caerulea* | Plantae | Tracheophyta | 26 | 105 | -0.02266 | 0.0074 | -0.3544 | 0.18062 |
| *Phylloscopus trochilus* | Animalia | Chordata | 8 | 95 | -0.00274 | 0.00564 | -0.45297 | 0.11351 |
| *Physcia aipolia* | Fungi | Ascomycota | 3 | 52 | 0.02946 | 0.005 | -1.4995 | 1.01986 |
| *Pica pica* | Animalia | Chordata | 6 | 90 | -0.00072 | 0.00296 | -0.10739 | 0.12703 |
| *Pilosella floribunda* | Plantae | Tracheophyta | 24 | 101 | -0.00307 | 0.00532 | -0.15246 | 0.1237 |
| *Pilosella lactucella* | Plantae | Tracheophyta | 5 | 67 | 0.00533 | 0.01166 | -0.62408 | 0.7481 |
| *Pimpinella saxifraga* | Plantae | Tracheophyta | 29 | 70 | 0.00439 | 0.00603 | -0.27339 | 0.10525 |
| *Pinguicula vulgaris* | Plantae | Tracheophyta | 29 | 104 | -0.01679 | 0.00712 | -0.32755 | 0.13539 |
| *Pinicola enucleator* | Animalia | Chordata | 3 | 52 | -0.01982 | 0.00043 | -0.38621 | 0.10583 |
| *Pinus uncinata* | Plantae | Tracheophyta | 3 | 70 | -0.00149 | 0.00178 | 0.05299 | 0.04515 |
| *Plagiobryum zierii* | Plantae | Bryophyta | 37 | 118 | 0.0064 | 0.0035 | 0.25223 | 0.09661 |
| *Plagiomnium undulatum* | Plantae | Bryophyta | 6 | 54 | -0.01723 | 0.01474 | 0.23443 | 0.23875 |
| *Plagiothecium cavifolium* | Plantae | Bryophyta | 6 | 73 | 0.00778 | 0.00467 | 0.09855 | 0.14134 |
| *Plagiothecium denticulatum* | Plantae | Bryophyta | 4 | 69 | 0.00739 | 0.01391 | 0.05933 | 0.49543 |
| *Plagiothecium undulatum* | Plantae | Bryophyta | 11 | 68 | -0.00949 | 0.00915 | -0.04053 | 0.22662 |
| *Plantago lanceolata* | Plantae | Tracheophyta | 33 | 84 | 0.01188 | 0.00432 | -0.00299 | 0.09864 |
| *Platambus maculatus* | Animalia | Arthropoda | 6 | 51 | 0.01032 | 0.01251 | 0.08 | 0.24138 |
| *Platanthera bifolia* | Plantae | Tracheophyta | 33 | 61 | 0.0063 | 0.00526 | 0.05447 | 0.09512 |
| *Platanthera chlorantha* | Plantae | Tracheophyta | 6 | 64 | -0.00163 | 0.00286 | -0.01401 | 0.08872 |
| *Pleurozium schreberi* | Plantae | Bryophyta | 7 | 58 | 0.00736 | 0.01556 | 0.46849 | 0.28742 |
| *Poa alpina* | Plantae | Tracheophyta | 52 | 84 | 0.00218 | 0.00547 | -0.20533 | 0.13453 |
| *Poa annua* | Plantae | Tracheophyta | 34 | 61 | -0.01265 | 0.00748 | -0.29479 | 0.12321 |
| *Poa arctica* | Plantae | Tracheophyta | 5 | 51 | -0.0006 | 0.00181 | 0.06457 | 0.01413 |
| *Poa flexuosa* | Plantae | Tracheophyta | 13 | 61 | -0.00775 | 0.00964 | -0.36561 | 0.27814 |
| *Poa glauca* | Plantae | Tracheophyta | 41 | 91 | 0.00169 | 0.00532 | -0.3253 | 0.13569 |
| *Poa nemoralis* | Plantae | Tracheophyta | 39 | 66 | -0.00022 | 0.00579 | -0.27876 | 0.1156 |
| *Poa pratensis* | Plantae | Tracheophyta | 39 | 70 | -0.00743 | 0.00602 | -0.36133 | 0.11591 |
| *Poa trivialis* | Plantae | Tracheophyta | 30 | 69 | 0.00079 | 0.00454 | -0.22125 | 0.11122 |
| *Pohlia cruda* | Plantae | Bryophyta | 7 | 102 | 0.01147 | 0.0091 | 0.22628 | 0.31624 |
| *Pohlia drummondii* | Plantae | Bryophyta | 3 | 76 | -0.00074 | 0.00457 | -0.08903 | 0.13447 |
| *Pohlia elongata* | Plantae | Bryophyta | 6 | 71 | -0.00919 | 0.01153 | -0.09817 | 0.30037 |
| *Pohlia filum* | Plantae | Bryophyta | 3 | 71 | -0.0032 | 0.00965 | -0.42883 | 0.15566 |
| *Polycentropus flavomaculatus* | Animalia | Arthropoda | 46 | 56 | -0.0087 | 0.00492 | -0.04016 | 0.07872 |
| *Polygala vulgaris* | Plantae | Tracheophyta | 20 | 74 | 0.01086 | 0.0059 | 0.12366 | 0.14573 |
| *Polygonatum odoratum* | Plantae | Tracheophyta | 11 | 58 | 0.00057 | 0.00503 | -0.07571 | 0.09722 |
| *Polygonatum verticillatum* | Plantae | Tracheophyta | 39 | 68 | 0.00914 | 0.0049 | -0.08483 | 0.10749 |
| *Polystichum braunii* | Plantae | Tracheophyta | 14 | 69 | 0.00436 | 0.01044 | -0.20429 | 0.19782 |
| *Polystichum lonchitis* | Plantae | Tracheophyta | 39 | 78 | -0.00505 | 0.00486 | -0.1452 | 0.10571 |
| *Polytrichastrum alpinum* | Plantae | Bryophyta | 7 | 80 | 0.00801 | 0.00778 | 0.36723 | 0.31016 |
| *Polytrichum commune* | Plantae | Bryophyta | 3 | 82 | -0.00118 | 0.00628 | 0.16817 | 0.29666 |
| *Polytrichum formosum* | Plantae | Bryophyta | 6 | 98 | 0.00843 | 0.00327 | 0.24474 | 0.0898 |
| *Polytrichum hyperboreum* | Plantae | Bryophyta | 2 | 69 | -0.00096 | NA | -0.13015 | NA |
| *Polytrichum longisetum* | Plantae | Bryophyta | 3 | 73 | 0.01456 | 0.00931 | 0.00995 | 1.31513 |
| *Porella cordaeana* | Plantae | Marchantiophyta | 5 | 108 | 0.01037 | 0.00302 | 0.29481 | 0.09715 |
| *Porella platyphylla* | Plantae | Marchantiophyta | 2 | 95 | 0.00855 | NA | 0.54401 | NA |
| *Potamogeton alpinus* | Plantae | Tracheophyta | 19 | 94 | 0.00739 | 0.00715 | 0.03054 | 0.1622 |
| *Potamogeton gramineus* | Plantae | Tracheophyta | 17 | 67 | 0.0012 | 0.0095 | 0.15987 | 0.16782 |
| *Potamogeton natans* | Plantae | Tracheophyta | 19 | 59 | 0.01688 | 0.00752 | 0.01858 | 0.17217 |
| *Potamogeton perfoliatus* | Plantae | Tracheophyta | 8 | 68 | 0.00975 | 0.005 | 0.0922 | 0.13236 |
| *Potamophylax latipennis* | Animalia | Arthropoda | 15 | 64 | -0.00395 | 0.00708 | -0.00492 | 0.12068 |
| *Potentilla argentea* | Plantae | Tracheophyta | 18 | 68 | -0.00262 | 0.00299 | -0.13305 | 0.06923 |
| *Potentilla crantzii* | Plantae | Tracheophyta | 48 | 80 | 0.00262 | 0.00523 | -0.17246 | 0.11379 |
| *Potentilla erecta* | Plantae | Tracheophyta | 34 | 104 | -0.0126 | 0.00609 | -0.28026 | 0.1176 |
| *Potentilla verna* | Plantae | Tracheophyta | 66 | 102 | 0.00198 | 0.00083 | -0.00086 | 0.02391 |
| *Primula acaulis* | Plantae | Tracheophyta | 16 | 57 | -0.00392 | 0.0109 | 0.0218 | 0.21557 |
| *Primula scandinavica* | Plantae | Tracheophyta | 18 | 77 | -0.00775 | 0.00362 | -0.08002 | 0.10462 |
| *Primula stricta* | Plantae | Tracheophyta | 4 | 54 | 0.00578 | 0.00441 | 0.13814 | 0.06623 |
| *Primula veris* | Plantae | Tracheophyta | 9 | 68 | 0.00129 | 0.00171 | -0.0174 | 0.04123 |
| *Procloeon bifidum* | Animalia | Arthropoda | 4 | 56 | -0.00083 | 0.01392 | 0.12563 | 0.3077 |
| *Proteinus brachypterus* | Animalia | Arthropoda | 2 | 53 | 0.02027 | NA | -1.61596 | NA |
| *Prunella modularis* | Animalia | Chordata | 2 | 52 | -0.00809 | NA | -0.23318 | NA |
| *Prunella vulgaris* | Plantae | Tracheophyta | 27 | 57 | -0.00448 | 0.00818 | -0.22599 | 0.11572 |
| *Pseudathyrium alpestre* | Plantae | Tracheophyta | 78 | 117 | 0.0028 | 0.00324 | -0.10702 | 0.08956 |
| *Pseudocalliergon trifarium* | Plantae | Bryophyta | 22 | 113 | 0.01068 | 0.00528 | 0.04596 | 0.15854 |
| *Pseudocyphellaria citrina* | Fungi | Ascomycota | 12 | 73 | -0.00656 | 0.00545 | -0.11477 | 0.12062 |
| *Pseudoleskeella nervosa* | Plantae | Bryophyta | 7 | 105 | 0.0082 | 0.00459 | 0.22473 | 0.13062 |
| *Pseudorchis straminea* | Plantae | Tracheophyta | 9 | 59 | 0.00842 | 0.01208 | -0.37124 | 0.23642 |
| *Puncturella noachina* | Animalia | Mollusca | 6 | 63 | 0.00154 | 0.00169 | 0.10295 | 0.03465 |
| *Pycnoporus cinnabarinus* | Fungi | Basidiomycota | 11 | 56 | 0.00712 | 0.01004 | -0.13193 | 0.25539 |
| *Pyrola minor* | Plantae | Tracheophyta | 45 | 105 | -0.00343 | 0.00549 | -0.30371 | 0.11669 |
| *Pyrrhula pyrrhula* | Animalia | Chordata | 13 | 49 | 0.0033 | 0.00474 | -0.03353 | 0.10251 |
| *Racomitrium ericoides* | Plantae | Bryophyta | 8 | 73 | -0.0018 | 0.00665 | -0.23853 | 0.11718 |
| *Racomitrium fasciculare* | Plantae | Bryophyta | 4 | 89 | 0.00726 | 0.00858 | 0.14791 | 0.31158 |
| *Racomitrium lanuginosum* | Plantae | Bryophyta | 11 | 76 | 0.01366 | 0.00703 | 0.40888 | 0.1183 |
| *Ranunculus auricomus* | Plantae | Tracheophyta | 26 | 56 | 0.02127 | 0.00674 | -0.09446 | 0.16609 |
| *Ranunculus flammula* | Plantae | Tracheophyta | 10 | 52 | 0.01213 | 0.01014 | -0.13542 | 0.20322 |
| *Ranunculus glacialis* | Plantae | Tracheophyta | 10 | 58 | -0.0148 | 0.01017 | 0.0568 | 0.39072 |
| *Ranunculus platanifolius* | Plantae | Tracheophyta | 24 | 104 | 0.00184 | 0.00581 | -0.14425 | 0.12602 |
| *Ranunculus propinquus* | Plantae | Tracheophyta | 52 | 108 | 0.00337 | 0.00312 | 0.01032 | 0.09641 |
| *Ranunculus pygmaeus* | Plantae | Tracheophyta | 11 | 52 | -0.0087 | 0.01943 | -0.28451 | 0.34359 |
| *Ranunculus reptans* | Plantae | Tracheophyta | 27 | 93 | -0.0004 | 0.00597 | -0.08312 | 0.11728 |
| *Regulus regulus* | Animalia | Chordata | 3 | 66 | 0.00888 | 0.00101 | 0.24913 | 0.07043 |
| *Rhacodiopsis rupestris* | Fungi | Ascomycota | 7 | 85 | 0.01576 | 0.01101 | 0.48559 | 0.36552 |
| *Rhinanthus groenlandicus* | Plantae | Tracheophyta | 46 | 109 | -0.00057 | 0.00488 | -0.01969 | 0.14362 |
| *Rhinanthus minor* | Plantae | Tracheophyta | 39 | 69 | -0.00427 | 0.00671 | -0.22545 | 0.13621 |
| *Rhizomnium pseudopunctatum* | Plantae | Bryophyta | 6 | 106 | 0.01557 | 0.00994 | 0.40936 | 0.32133 |
| *Rhizomnium punctatum* | Plantae | Bryophyta | 4 | 61 | -0.00867 | 0.01766 | 0.16868 | 0.439 |
| *Rhodiola rosea* | Plantae | Tracheophyta | 33 | 61 | -0.0114 | 0.00751 | -0.38621 | 0.13068 |
| *Rhodofomes roseus* | Fungi | Basidiomycota | 3 | 62 | 0.01318 | 0.01217 | 0.53946 | 0.12342 |
| *Rhyacophila nubila* | Animalia | Arthropoda | 45 | 56 | -0.00488 | 0.00665 | 0.1066 | 0.09228 |
| *Rhynchospora alba* | Plantae | Tracheophyta | 16 | 66 | -0.0035 | 0.00695 | -0.01412 | 0.14329 |
| *Rhytidiadelphus loreus* | Plantae | Bryophyta | 14 | 72 | -0.00145 | 0.00788 | 0.10395 | 0.16729 |
| *Rhytidiadelphus triquetrus* | Plantae | Bryophyta | 5 | 61 | -0.0106 | 0.01519 | 0.12719 | 0.32487 |
| *Riparia riparia* | Animalia | Chordata | 6 | 95 | -0.00713 | 0.0039 | -0.21412 | 0.11565 |
| *Rosa majalis* | Plantae | Tracheophyta | 21 | 58 | 0.00623 | 0.00421 | 0.30298 | 0.07946 |
| *Rosa mollis* | Plantae | Tracheophyta | 11 | 81 | -0.00366 | 0.0073 | -0.10492 | 0.20706 |
| *Rubus nessensis* | Plantae | Tracheophyta | 4 | 57 | 0.00002 | 0.00324 | -0.01595 | 0.08593 |
| *Rumex acetosa* | Plantae | Tracheophyta | 36 | 104 | -0.01434 | 0.00635 | -0.27032 | 0.12765 |
| *Rumex crispus* | Plantae | Tracheophyta | 9 | 64 | 0.01805 | 0.01202 | -0.16293 | 0.26457 |
| *Rumex lapponicus* | Plantae | Tracheophyta | 22 | 90 | 0.01418 | 0.00628 | 0.34913 | 0.20361 |
| *Rumex obtusifolius* | Plantae | Tracheophyta | 3 | 50 | 0.00139 | 0.00785 | 0.1278 | 0.10516 |
| *Sabulina stricta* | Plantae | Tracheophyta | 14 | 63 | 0.01067 | 0.00939 | -0.07698 | 0.18118 |
| *Sagina procumbens* | Plantae | Tracheophyta | 30 | 54 | -0.00494 | 0.0082 | -0.14903 | 0.1291 |
| *Sagina saginoides* | Plantae | Tracheophyta | 22 | 60 | 0.00603 | 0.01224 | -0.36239 | 0.22742 |
| *Salicornia europaea* | Plantae | Tracheophyta | 9 | 53 | 0.0201 | 0.0122 | 0.01208 | 0.29062 |
| *Salix arbuscula* | Plantae | Tracheophyta | 22 | 54 | -0.00012 | 0.0041 | -0.06405 | 0.07491 |
| *Salix aurita* | Plantae | Tracheophyta | 34 | 65 | 0.00254 | 0.00567 | -0.05438 | 0.10401 |
| *Salix borealis* | Plantae | Tracheophyta | 38 | 96 | -0.00543 | 0.00452 | -0.09692 | 0.10576 |
| *Salix caprea* | Plantae | Tracheophyta | 43 | 70 | -0.00412 | 0.00605 | -0.20111 | 0.11514 |
| *Salix glauca* | Plantae | Tracheophyta | 37 | 74 | -0.00466 | 0.0075 | -0.23059 | 0.15655 |
| *Salix hastata* | Plantae | Tracheophyta | 39 | 78 | 0.00147 | 0.00732 | -0.28841 | 0.16909 |
| *Salix herbacea* | Plantae | Tracheophyta | 32 | 105 | -0.01807 | 0.00725 | -0.38278 | 0.16437 |
| *Salix lanata* | Plantae | Tracheophyta | 30 | 58 | 0.00347 | 0.00955 | -0.25972 | 0.18233 |
| *Salix lapponum* | Plantae | Tracheophyta | 44 | 59 | -0.00645 | 0.00681 | -0.24336 | 0.14288 |
| *Salix myrsinifolia* | Plantae | Tracheophyta | 50 | 94 | 0.0042 | 0.00441 | -0.16978 | 0.0938 |
| *Salix myrsinites* | Plantae | Tracheophyta | 29 | 66 | 0.00617 | 0.00916 | -0.24169 | 0.1949 |
| *Salix pentandra* | Plantae | Tracheophyta | 40 | 64 | 0.01075 | 0.00443 | 0.07081 | 0.08674 |
| *Salix phylicifolia* | Plantae | Tracheophyta | 39 | 67 | 0.00725 | 0.00813 | -0.18235 | 0.16585 |
| *Salix polaris* | Plantae | Tracheophyta | 11 | 63 | -0.00701 | 0.00132 | -0.11111 | 0.04625 |
| *Salix reticulata* | Plantae | Tracheophyta | 25 | 60 | -0.02881 | 0.01076 | -0.30909 | 0.25104 |
| *Salix triandra* | Plantae | Tracheophyta | 9 | 51 | 0.01905 | 0.00614 | -0.00565 | 0.1728 |
| *Sanicula europaea* | Plantae | Tracheophyta | 9 | 71 | -0.00657 | 0.00731 | -0.25594 | 0.20086 |
| *Sanionia uncinata* | Plantae | Bryophyta | 16 | 66 | 0.00535 | 0.01189 | 0.17327 | 0.26252 |
| *Sarmentypnum exannulatum* | Plantae | Bryophyta | 9 | 78 | 0.01657 | 0.01139 | 0.54423 | 0.25977 |
| *Sarmentypnum sarmentosum* | Plantae | Bryophyta | 4 | 68 | 0.0087 | 0.00364 | 0.14776 | 0.19275 |
| *Saussurea alpina* | Plantae | Tracheophyta | 36 | 61 | -0.02662 | 0.00765 | -0.42065 | 0.15974 |
| *Saxifraga aizoides* | Plantae | Tracheophyta | 42 | 117 | -0.00587 | 0.00496 | -0.26571 | 0.10377 |
| *Saxifraga cespitosa* | Plantae | Tracheophyta | 13 | 55 | 0.01574 | 0.01873 | -0.27349 | 0.31419 |
| *Saxifraga cotyledon* | Plantae | Tracheophyta | 33 | 55 | -0.0032 | 0.00487 | -0.3112 | 0.08111 |
| *Saxifraga oppositifolia* | Plantae | Tracheophyta | 40 | 118 | 0.00131 | 0.00456 | -0.08278 | 0.0994 |
| *Saxifraga rivularis* | Plantae | Tracheophyta | 16 | 62 | 0.00139 | 0.01315 | -0.27408 | 0.25183 |
| *Saxifraga tridactylites* | Plantae | Tracheophyta | 4 | 67 | -0.00597 | 0.00477 | -0.13771 | 0.1758 |
| *Scapania aspera* | Plantae | Marchantiophyta | 4 | 107 | -0.00018 | 0.00376 | 0.06538 | 0.09132 |
| *Scapania curta* | Plantae | Marchantiophyta | 4 | 78 | 0.00293 | 0.00843 | 1.24476 | 1.3092 |
| *Scapania uliginosa* | Plantae | Marchantiophyta | 2 | 61 | 0.00594 | NA | 0.64586 | NA |
| *Scapania umbrosa* | Plantae | Marchantiophyta | 16 | 99 | 0.01226 | 0.0041 | 0.20905 | 0.10737 |
| *Scapania undulata* | Plantae | Marchantiophyta | 9 | 111 | 0.00799 | 0.00405 | 0.38116 | 0.08951 |
| *Scaphander lignarius* | Animalia | Mollusca | 4 | 50 | -0.01117 | 0.00042 | -0.28225 | 0.06704 |
| *Scheuchzeria palustris* | Plantae | Tracheophyta | 24 | 71 | -0.00521 | 0.00725 | -0.16581 | 0.13864 |
| *Schistidium maritimum* | Plantae | Bryophyta | 2 | 97 | 0.0002 | NA | 0.00739 | NA |
| *Schistidium papillosum* | Plantae | Bryophyta | 15 | 96 | 0.01813 | 0.00537 | 0.27462 | 0.16721 |
| *Schistochilopsis incisa* | Plantae | Marchantiophyta | 8 | 102 | 0.01803 | 0.00461 | 0.45692 | 0.21923 |
| *Schistostega pennata* | Plantae | Bryophyta | 3 | 89 | 0.01511 | 0.00018 | 0.57113 | 0.1342 |
| *Schljakovianthus quadrilobus* | Plantae | Marchantiophyta | 4 | 73 | 0.0134 | 0.01053 | -0.18663 | 0.53762 |
| *Schoenus ferrugineus* | Plantae | Tracheophyta | 17 | 71 | 0.0025 | 0.00546 | -0.05565 | 0.11092 |
| *Scirpus sylvaticus* | Plantae | Tracheophyta | 15 | 58 | 0.01333 | 0.00641 | 0.27489 | 0.14465 |
| *Sciuro-hypnum plumosum* | Plantae | Bryophyta | 4 | 61 | -0.00562 | 0.02805 | 0.02062 | 0.61786 |
| *Scolopax rusticola* | Animalia | Chordata | 2 | 49 | 0.001 | NA | 0.06623 | NA |
| *Scorpidium revolvens* | Plantae | Bryophyta | 7 | 101 | 0.01109 | 0.0073 | 0.306 | 0.23369 |
| *Scorzoneroides autumnalis* | Plantae | Tracheophyta | 7 | 79 | -0.00379 | 0.00586 | -0.33953 | 0.19702 |
| *Scrophularia nodosa* | Plantae | Tracheophyta | 21 | 54 | 0.0024 | 0.00782 | -0.15889 | 0.12937 |
| *Scutellaria galericulata* | Plantae | Tracheophyta | 15 | 49 | 0.00636 | 0.00919 | 0.05179 | 0.13204 |
| *Sedum anglicum* | Plantae | Tracheophyta | 6 | 51 | 0.00533 | 0.00248 | -0.0714 | 0.06678 |
| *Sedum annuum* | Plantae | Tracheophyta | 23 | 54 | -0.00612 | 0.00746 | -0.31447 | 0.10225 |
| *Selaginella selaginoides* | Plantae | Tracheophyta | 37 | 79 | 0.0094 | 0.00592 | 0.03917 | 0.11671 |
| *Seligeria diversifolia* | Plantae | Bryophyta | 10 | 109 | 0.00156 | 0.00674 | 0.14017 | 0.13335 |
| *Seligeria recurvata* | Plantae | Bryophyta | 13 | 109 | 0.00324 | 0.00404 | 0.00781 | 0.10414 |
| *Sialis lutaria* | Animalia | Arthropoda | 7 | 53 | 0.00166 | 0.00129 | 0.02219 | 0.02368 |
| *Sibbaldia procumbens* | Plantae | Tracheophyta | 22 | 60 | -0.03583 | 0.00947 | -0.37829 | 0.25122 |
| *Sigara distincta* | Animalia | Arthropoda | 15 | 49 | 0.00521 | 0.01508 | 0.04025 | 0.20544 |
| *Silene acaulis* | Plantae | Tracheophyta | 28 | 61 | -0.02563 | 0.00914 | -0.42959 | 0.16303 |
| *Silene flos-cuculi* | Plantae | Tracheophyta | 15 | 68 | 0.00788 | 0.00999 | -0.08934 | 0.19233 |
| *Silene suecica* | Plantae | Tracheophyta | 72 | 119 | 0.00521 | 0.00418 | -0.05867 | 0.12101 |
| *Silene vulgaris* | Plantae | Tracheophyta | 15 | 49 | -0.02981 | 0.01475 | -0.69516 | 0.15635 |
| *Siphonoperla burmeisteri* | Animalia | Arthropoda | 40 | 69 | -0.00867 | 0.00383 | -0.03971 | 0.05673 |
| *Siphula ceratites* | Fungi | Ascomycota | 4 | 52 | 0.02498 | 0.00559 | -0.18986 | 0.45237 |
| *Sitta europaea* | Animalia | Chordata | 3 | 62 | 0.00551 | 0.01104 | 0.64482 | 0.68665 |
| *Solanum lycopersicum* | Plantae | Tracheophyta | 8 | 79 | 0.00114 | 0.00034 | 0.03587 | 0.01266 |
| *Somateria mollissima* | Animalia | Chordata | 8 | 97 | -0.01947 | 0.00303 | -0.28993 | 0.27813 |
| *Sonchus arvensis* | Plantae | Tracheophyta | 13 | 64 | 0.01646 | 0.00916 | -0.22277 | 0.17788 |
| *Sorex araneus* | Animalia | Chordata | 6 | 99 | 0.01373 | 0.01002 | 0.621 | 0.44785 |
| *Sorex minutus* | Animalia | Chordata | 4 | 59 | 0.01018 | 0.0076 | -0.18443 | 0.29658 |
| *Sparganium angustifolium* | Plantae | Tracheophyta | 24 | 62 | 0.012 | 0.00778 | -0.09058 | 0.17587 |
| *Sparganium emersum* | Plantae | Tracheophyta | 2 | 64 | 0.00153 | NA | -0.13434 | NA |
| *Sparganium natans* | Plantae | Tracheophyta | 12 | 49 | 0.02894 | 0.01319 | 0.10199 | 0.25195 |
| *Spergularia marina* | Plantae | Tracheophyta | 56 | 113 | 0.0075 | 0.00222 | 0.0734 | 0.0674 |
| *Sphagnum angustifolium* | Plantae | Bryophyta | 22 | 55 | -0.00141 | 0.00654 | -0.18556 | 0.10448 |
| *Sphagnum balticum* | Plantae | Bryophyta | 14 | 51 | -0.0051 | 0.01158 | -0.1346 | 0.17549 |
| *Sphagnum capillifolium* | Plantae | Bryophyta | 21 | 106 | -0.0024 | 0.00472 | -0.09454 | 0.1166 |
| *Sphagnum compactum* | Plantae | Bryophyta | 15 | 113 | 0.01354 | 0.00441 | 0.1037 | 0.13455 |
| *Sphagnum divinum* | Plantae | Bryophyta | 7 | 53 | -0.0091 | 0.00629 | -0.22059 | 0.15688 |
| *Sphagnum fallax* | Plantae | Bryophyta | 25 | 51 | 0.00138 | 0.00709 | -0.13865 | 0.11558 |
| *Sphagnum flexuosum* | Plantae | Bryophyta | 17 | 82 | 0.00297 | 0.00682 | -0.16896 | 0.14584 |
| *Sphagnum fuscum* | Plantae | Bryophyta | 18 | 55 | -0.00679 | 0.00597 | -0.1185 | 0.08186 |
| *Sphagnum girgensohnii* | Plantae | Bryophyta | 18 | 107 | 0.00534 | 0.00627 | -0.1403 | 0.1306 |
| *Sphagnum isoviitae* | Plantae | Bryophyta | 32 | 52 | 0.00041 | 0.00701 | -0.13974 | 0.10484 |
| *Sphagnum lindbergii* | Plantae | Bryophyta | 18 | 113 | 0.00459 | 0.00644 | -0.10668 | 0.16197 |
| *Sphagnum papillosum* | Plantae | Bryophyta | 35 | 106 | 0.00326 | 0.0039 | -0.0395 | 0.0734 |
| *Sphagnum platyphyllum* | Plantae | Bryophyta | 9 | 50 | 0.00119 | 0.01621 | -0.09599 | 0.29002 |
| *Sphagnum riparium* | Plantae | Bryophyta | 10 | 110 | -0.00583 | 0.00518 | -0.23226 | 0.15533 |
| *Sphagnum rubellum* | Plantae | Bryophyta | 29 | 55 | 0.00268 | 0.00653 | -0.01172 | 0.10663 |
| *Sphagnum rubiginosum* | Plantae | Bryophyta | 12 | 100 | 0.0069 | 0.0052 | -0.06397 | 0.16879 |
| *Sphagnum russowii* | Plantae | Bryophyta | 22 | 112 | 0.00041 | 0.0062 | -0.15547 | 0.1105 |
| *Sphagnum squarrosum* | Plantae | Bryophyta | 9 | 52 | 0.00086 | 0.00886 | -0.03318 | 0.18699 |
| *Sphagnum subnitens* | Plantae | Bryophyta | 24 | 55 | -0.00332 | 0.00832 | -0.12335 | 0.1402 |
| *Sphagnum subsecundum* | Plantae | Bryophyta | 18 | 53 | -0.00903 | 0.00789 | -0.22831 | 0.14134 |
| *Sphagnum tenellum* | Plantae | Bryophyta | 20 | 106 | 0.00647 | 0.00393 | 0.09259 | 0.10116 |
| *Sphagnum teres* | Plantae | Bryophyta | 13 | 52 | 0.00924 | 0.00978 | -0.15722 | 0.16176 |
| *Sphagnum warnstorfii* | Plantae | Bryophyta | 19 | 117 | 0.00748 | 0.00584 | 0.02067 | 0.16186 |
| *Sphenolobus minutus* | Plantae | Marchantiophyta | 15 | 100 | 0.02345 | 0.00712 | 0.61811 | 0.20799 |
| *Stellaria crassifolia* | Plantae | Tracheophyta | 9 | 53 | 0.00618 | 0.02016 | -0.14429 | 0.3531 |
| *Stellaria graminea* | Plantae | Tracheophyta | 35 | 58 | -0.00573 | 0.00596 | -0.23855 | 0.10586 |
| *Stellaria longifolia* | Plantae | Tracheophyta | 13 | 55 | -0.00529 | 0.01165 | -0.36524 | 0.17998 |
| *Stellaria nemorum* | Plantae | Tracheophyta | 29 | 65 | -0.01355 | 0.00743 | -0.41947 | 0.11183 |
| *Stercorarius parasiticus* | Animalia | Chordata | 2 | 49 | -0.0336 | NA | -0.45256 | NA |
| *Stereocaulon vesuvianum* | Fungi | Ascomycota | 3 | 53 | 0.01614 | 0.00051 | 1.66852 | 2.63851 |
| *Steromphala cineraria* | Animalia | Mollusca | 21 | 102 | -0.00297 | 0.0026 | -0.10128 | 0.06404 |
| *Struthiopteris spicant* | Plantae | Tracheophyta | 44 | 70 | 0.0033 | 0.00539 | -0.07621 | 0.10882 |
| *Sturnus vulgaris* | Animalia | Chordata | 4 | 59 | -0.00604 | 0.00244 | -0.16764 | 0.2175 |
| *Suaeda maritima* | Plantae | Tracheophyta | 5 | 53 | 0.02488 | 0.01321 | 0.04325 | 0.37553 |
| *Succisa pratensis* | Plantae | Tracheophyta | 39 | 54 | -0.00296 | 0.00759 | -0.15024 | 0.12347 |
| *Syntrichia ruralis* | Plantae | Bryophyta | 8 | 106 | 0.00788 | 0.00549 | 0.3231 | 0.14416 |
| *Tachinus pallipes* | Animalia | Arthropoda | 3 | 62 | -0.001 | 0.00002 | 0.21626 | 0.03754 |
| *Tayloria lingulata* | Plantae | Bryophyta | 2 | 70 | -0.01314 | NA | -8.9534 | NA |
| *Tectura virginea* | Animalia | Mollusca | 14 | 102 | -0.00291 | 0.00301 | -0.05855 | 0.06716 |
| *Testudinalia testudinalis* | Animalia | Mollusca | 7 | 102 | -0.00231 | 0.0011 | -0.05681 | 0.03324 |
| *Tetralophozia setiformis* | Plantae | Marchantiophyta | 4 | 115 | 0.0059 | 0.00266 | 0.17253 | 0.0464 |
| *Tetrao urogallus* | Animalia | Chordata | 5 | 104 | -0.00791 | 0.00436 | -0.26318 | 0.08774 |
| *Tetraplodon mnioides* | Plantae | Bryophyta | 8 | 110 | 0.01895 | 0.00639 | 0.15045 | 0.38272 |
| *Thalictrum alpinum* | Plantae | Tracheophyta | 36 | 112 | -0.0048 | 0.00606 | -0.21776 | 0.14023 |
| *Thalictrum flavum* | Plantae | Tracheophyta | 22 | 60 | -0.00588 | 0.0071 | -0.05638 | 0.13621 |
| *Timmia bavarica* | Plantae | Bryophyta | 2 | 67 | -0.00551 | NA | -0.83211 | NA |
| *Tofieldia pusilla* | Plantae | Tracheophyta | 37 | 106 | -0.01582 | 0.00546 | -0.17389 | 0.12457 |
| *Tortella tortuosa* | Plantae | Bryophyta | 25 | 70 | 0.00702 | 0.00719 | 0.31467 | 0.14051 |
| *Tortula subulata* | Plantae | Bryophyta | 3 | 98 | 0.00178 | 0.00006 | 0.07071 | 0.00512 |
| *Trichophorum cespitosum* | Plantae | Tracheophyta | 36 | 104 | -0.00169 | 0.00539 | -0.15771 | 0.11847 |
| *Triglochin palustris* | Plantae | Tracheophyta | 30 | 55 | 0.0124 | 0.00661 | -0.03142 | 0.10723 |
| *Trilophozia quinquedentata* | Plantae | Marchantiophyta | 14 | 108 | 0.01956 | 0.00594 | 0.4584 | 0.14878 |
| *Tripleurospermum subpolare* | Plantae | Tracheophyta | 22 | 71 | -0.00364 | 0.00608 | -0.13591 | 0.19976 |
| *Tritia incrassata* | Animalia | Mollusca | 4 | 102 | -0.00211 | 0.00016 | -0.04995 | 0.0104 |
| *Trollius europaeus* | Plantae | Tracheophyta | 11 | 54 | 0.0099 | 0.01061 | -0.10092 | 0.21033 |
| *Turdus iliacus* | Animalia | Chordata | 4 | 107 | -0.01768 | 0.00958 | -0.40403 | 0.29718 |
| *Turdus philomelos* | Animalia | Chordata | 2 | 96 | -0.00742 | NA | -0.33512 | NA |
| *Turdus pilaris* | Animalia | Chordata | 13 | 95 | -0.00778 | 0.00195 | -0.13633 | 0.12132 |
| *Turritella communis* | Animalia | Mollusca | 10 | 86 | -0.00173 | 0.00139 | -0.05345 | 0.0335 |
| *Udea lutealis* | Animalia | Arthropoda | 5 | 69 | -0.01207 | 0.00532 | -0.06637 | 0.18131 |
| *Ulmus glabra* | Plantae | Tracheophyta | 36 | 60 | 0.00262 | 0.00488 | -0.0647 | 0.10092 |
| *Ulota drummondii* | Plantae | Bryophyta | 4 | 99 | 0.00789 | 0.0023 | 0.21438 | 0.04104 |
| *Ulota phyllantha* | Plantae | Bryophyta | 2 | 101 | 0.00201 | NA | 0.13227 | NA |
| *Uria aalge* | Animalia | Chordata | 2 | 98 | -0.00948 | NA | -0.76368 | NA |
| *Usnea dasypoga* | Fungi | Ascomycota | 14 | 72 | 0.00597 | 0.00628 | -0.08674 | 0.14743 |
| *Utricularia intermedia* | Plantae | Tracheophyta | 17 | 64 | 0.01396 | 0.00524 | 0.15182 | 0.10914 |
| *Utricularia minor* | Plantae | Tracheophyta | 11 | 61 | 0.02521 | 0.01144 | 0.1633 | 0.24296 |
| *Utricularia vulgaris* | Plantae | Tracheophyta | 5 | 63 | 0.00681 | 0.01884 | -0.46664 | 0.34675 |
| *Vaccinium microcarpum* | Plantae | Tracheophyta | 65 | 101 | 0.00503 | 0.00433 | -0.0467 | 0.113 |
| *Vaccinium myrtillus* | Plantae | Tracheophyta | 31 | 104 | -0.01748 | 0.00719 | -0.3421 | 0.14334 |
| *Vaccinium oxycoccos* | Plantae | Tracheophyta | 58 | 116 | 0.00164 | 0.00275 | 0.01224 | 0.08068 |
| *Vaccinium uliginosum* | Plantae | Tracheophyta | 31 | 104 | -0.02036 | 0.00725 | -0.29947 | 0.14667 |
| *Valeriana excelsa* | Plantae | Tracheophyta | 76 | 117 | 0.00408 | 0.0029 | -0.11418 | 0.08309 |
| *Veronica alpina* | Plantae | Tracheophyta | 29 | 67 | -0.01752 | 0.00933 | -0.3163 | 0.17269 |
| *Veronica arvensis* | Plantae | Tracheophyta | 7 | 65 | 0.00116 | 0.0046 | 0.12481 | 0.13832 |
| *Veronica beccabunga* | Plantae | Tracheophyta | 12 | 58 | -0.00036 | 0.00271 | 0.04191 | 0.0602 |
| *Veronica fruticans* | Plantae | Tracheophyta | 20 | 63 | -0.01228 | 0.01089 | -0.40342 | 0.2016 |
| *Veronica scutellata* | Plantae | Tracheophyta | 14 | 60 | 0.00247 | 0.00651 | -0.06736 | 0.11284 |
| *Veronica serpyllifolia* | Plantae | Tracheophyta | 26 | 53 | -0.02766 | 0.00835 | -0.28811 | 0.14817 |
| *Viburnum opulus* | Plantae | Tracheophyta | 21 | 58 | -0.00586 | 0.0068 | -0.16848 | 0.14393 |
| *Vicia sepium* | Plantae | Tracheophyta | 33 | 51 | -0.00529 | 0.00787 | -0.2949 | 0.11595 |
| *Vicia sylvatica* | Plantae | Tracheophyta | 34 | 55 | 0.00335 | 0.00618 | -0.17812 | 0.10812 |
| *Viola biflora* | Plantae | Tracheophyta | 34 | 106 | -0.01338 | 0.00632 | -0.22083 | 0.13033 |
| *Viola canina* | Plantae | Tracheophyta | 49 | 80 | 0.00813 | 0.00413 | -0.0785 | 0.09669 |
| *Viola epipsila* | Plantae | Tracheophyta | 8 | 56 | -0.00694 | 0.01132 | -0.42788 | 0.19944 |
| *Viola mirabilis* | Plantae | Tracheophyta | 28 | 70 | -0.00352 | 0.00585 | -0.17588 | 0.11267 |
| *Viola palustris* | Plantae | Tracheophyta | 38 | 111 | -0.0153 | 0.00581 | -0.25891 | 0.11617 |
| *Viola riviniana* | Plantae | Tracheophyta | 50 | 83 | 0.00606 | 0.00398 | -0.22316 | 0.09431 |
| *Volvopluteus gloiocephalus* | Fungi | Basidiomycota | 2 | 66 | 0.0123 | NA | 0.4796 | NA |
| *Woodsia alpina* | Plantae | Tracheophyta | 20 | 62 | -0.00905 | 0.00887 | 0.04236 | 0.1571 |
| *Woodsia ilvensis* | Plantae | Tracheophyta | 28 | 66 | 0.00136 | 0.0054 | -0.20167 | 0.10159 |
| *Xanthorhoe decoloraria* | Animalia | Arthropoda | 6 | 58 | -0.01634 | 0.01962 | -0.60576 | 0.29016 |
